# Settling time of a vibrational wavepacket in ionization

**DOI:** 10.1038/ncomms9197

**Published:** 2015-09-01

**Authors:** Yasuo Nabekawa, Yusuke Furukawa, Tomoya Okino, A. Amani Eilanlou, Eiji J. Takahashi, Kaoru Yamanouchi, Katsumi Midorikawa

**Affiliations:** 1 Attosecond Science Research Team, RIKEN Center for Advanced Photonics (RAP), 2-1 Hirosawa, Wako-shi, Saitama 351-0198, Japan; 2 Department of Chemistry, School of Science, The University of Tokyo, 7-3-1 Hongo, Bunkyo, Tokyo 113-0033, Japan

## Abstract

The vibrational wavepacket of a diatomic molecular ion at the time of ionization is usually considered to be generated on the basis of the Franck–Condon principle. According to this principle, the amplitude of each vibrational wavefunction in the wavepacket is given by the overlap integral between each vibrational wavefunction and the ground vibrational wavefunction in the neutral molecule, and hence, the amplitude should be a real number, or equivalently, a complex number the phase of which is equal to zero. Here we report the observation of a non-trivial phase modulation of the amplitudes of vibrational wavefunctions in a wavepacket generated in the ground electronic state of a 
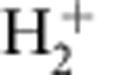
 molecular ion at the time of ionization. The phase modulation results in a group delay of the specific vibrational states of order 1 fs, which can be regarded as the settling time required to compose the initial vibrational wavepacket.

## Abstract (web summary)

Instantaneous generation of a vibrational wavepacket in a molecular ion is usually assumed in an ionization process. Here, by means of frequency-resolved optical gating, the authors observe a non-trivial phase modulation in a H_2_^+^ molecule, which is interpreted as a ∼1-fs settling time.

## Graphical abstract

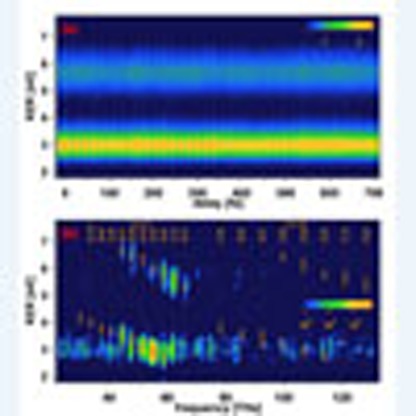

The 
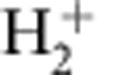
 molecular ion has been widely investigated to obtain essential benchmarks in molecular physics because it has the simplest structure among molecules. The real-time observations of a vibrational wavepacket of 
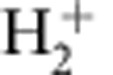
 using an a-few-cycle laser pulse^1^,^2^, an attosecond high-harmonic pulse^3^, and an X-ray free-electron laser pulse^4^ are examples of such fundamental studies. In these studies, a vibrational wavepacket in the ground bound state (1*sσ*_*g*_) of 
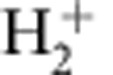
 is created by the ionization of H_2_ with a pump pulse. Then, after a time delay, the 
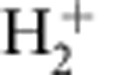
 is excited to the repulsive state of 
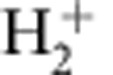
 or further ionized into the doubly charged state by the irradiation of a second probe pulse. The real-time motion of the wavepacket can be tracked by measuring the ion fragments of H^+^ or detached electrons by scanning the delay of the probe pulse.

In general, a vibrational wavepacket in the 1*sσ*_*g*_ state of 
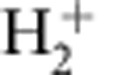
 at time *t*, *ϕ*^*g*^(*R*;*t*), is expressed as a coherent superposition of vibrational wavefunctions, 
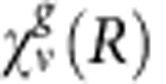
, with the time-evolving phase factor of 
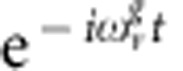
, namely, 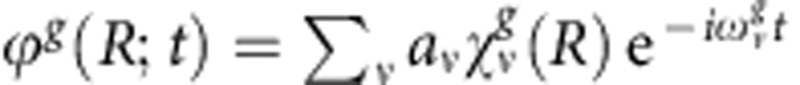 (ref. 2), where *ν* is the vibrational quantum number and the internuclear distance and the amplitude of the *ν*th vibrational wavefunction are denoted as *R* and *a*_*ν*_, respectively. The eigenenergy of the *ν*th vibrational state coincides with 
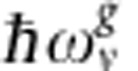
. The constant of proportionality, *ħ*, is Planck's constant divided by 2*π*. Although the amplitude, *a*_*ν*_, can generally be a complex number^1^,^5^, we commonly substitute 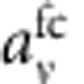 for *a*_*ν*_ as a good approximation for the situation of an ultrafast ionization process, where 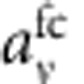 is obtained from an overlap integral of 
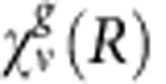
 and 
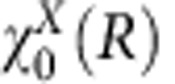
 as 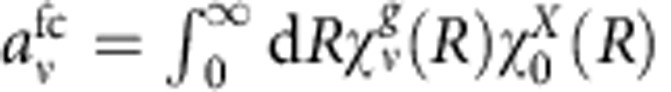, where 
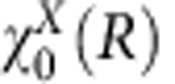
 is the ground vibrational wavefunction in the 
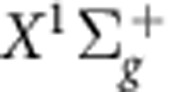
 state of a neutral H_2_ molecule. A detailed theoretical model considering the electronic transition moment can provide us with a more accurate *a*_*ν*_, and then, the resultant magnitude correction for *a*_*ν*_ can be used to prove the consistency of the experimental data^6^. Magnitude correction is also necessary when 
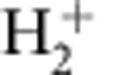
 is generated via tunnelling ionization with an intense near-infrared laser pulse^5^,^7^. In contrast, the phase of *a*_*ν*_ at ionization has been omitted, or at least, it has not yet been necessary to consider it to explain the experimental data related to the ionization of H_2_.

In this paper, we demonstrate a non-trivial phase modulation of *a*_*ν*_, measured by applying the frequency-resolved optical gating (FROG)^8^ technique to the two-dimensional delay-energy spectrogram of H^+^ fragment ions^9^. This is the first measurement, to the best of our knowledge, of the phase at the time of birth of a vibrational wavepacket. We have found that the phase modulation is caused by the interference of the phase in the wavefunction of a continuum electron ionized by the one-photon absorption of an extreme ultraviolet (XUV) attosecond pulse train (APT).

## Results

### Experimental data

**Figure 1:**
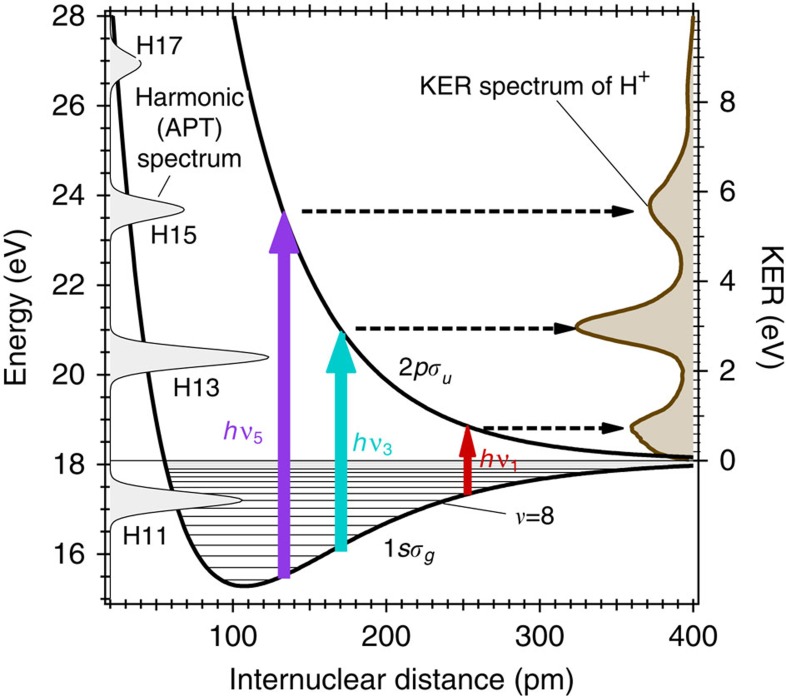
Experimental scheme. The relevant adiabatic potential energy curves (1*sσ*_*g*_ and 2*pσ*_*u*_) of 
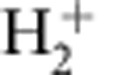
 are shown as solid black curves. A typical KER spectrum of a H^+^ ion is depicted as a brown curve with a shaded area on the right-hand side. The harmonic spectrum ranging from the 11th to 17th orders in the pump APT to ionize neutral H_2_ is shown as a grey curve with shaded areas. The energy on the left axis, indicating the photon energy of the high-harmonic and the energy of the adiabatic potential, is measured from the ground vibrational state of the 
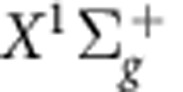
 electronic state of H_2_. The vibrational wavepacket created in the 1*sσ*_*g*_ state is probed by one-photon excitation to the 2*pσ*_*u*_ state by absorbing the 3rd- and 5th-order harmonic components in the probe APT, which are indicated by light blue and violet arrows, respectively. The weak fundamental component, depicted as a red arrow, also excites the 1*sσ*_*g*_ state, while this component was not used to retrieve the vibrational wavepacket amplitude.

**Figure 2:**
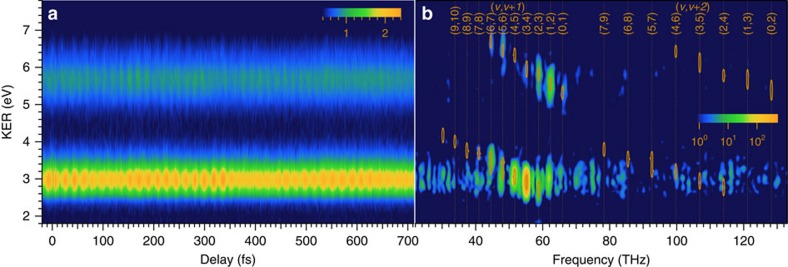
Experimental result. (**a**) Delay-KER spectrogram of H^+^ fragments measured using a velocity mapping imaging (VMI) spectrometer. (**b**) Magnitude square of the Fourier transform of the delay-KER spectrogram in Fig. 2a. The color scale in (**a**) changes linearly with the intensity, while a logarithmic scale is used to plot the intensity in (**b**) to clearly exhibit the beat frequency components. Pairs of numbers assigning vibrational states as beat frequency sources are depicted at the top of this figure. We also depict the bandpass filter used to obtain the target delay-KER spectrogram, shown in Fig. 3a, as contour plots (ellipses).

We show a schematic of the energy diagram for the generation and observation of the 
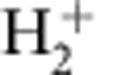
 (or 
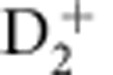
) vibrational wavepacket adopted in our experiment in Fig. 1. The spectrum of an APT^10^,^11^ used to ionize H_2_ is shown in Supplementary Fig. 1. In our experiment, a vibrational wavepacket in the 1*sσ*_*g*_ state is first created via the ionization of H_2_ by absorbing one photon in a pump pulse composed of the harmonic spectrum ranging from the 11th to 21st orders. Then, the 
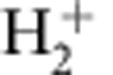
 molecule dissociates by excitation from the 1*sσ*_*g*_ state to the repulsive 2*pσ*_*u*_ state by the absorption of another photon in the probe pulse accompanying the 3rd and 5th components (represented as light blue and violet arrows in Fig. 1, respectively), resulting in the kinetic energy release (KER) spectrum of H^+^ fragments exhibiting two peaks at ∼3 and ∼5.7 eV, shown as a light brown curve with a shaded area on the right-hand side of Fig. 1. Another peak at ∼0.8 eV is ascribed to the excitation by absorbing the fundamental photon. We obtained the two-dimensional spectrogram of H^+^ fragments shown in Fig. 2a by scanning the delay of the probe pulse. The experimental^12^,^13^ detail and the reason why we disregard the KER spectrum at low KER region are described in Supplementary Note 1.

### Phase retrieval of vibrational wavefunction

The first aim of our study is to extract the phase factor from the measured delay-KER spectrogram in Fig. 2a. Thus, we adopted a physical model that suitably expresses the excitation process from the 1*sσ*_*g*_ to 2*pσ*_*u*_ states, which is based on the Hamiltonian of a two-level system interacting with an optical field via a dipole interaction^2^,^5^,^14^. This kind of Hamiltonian is widely used for analysing the vibrational wavepacket dynamics of a 
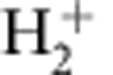
 molecular ion. By applying time-dependent perturbation theory to the Schrödinger equation with this Hamiltonian, we found the one-photon transition amplitude from the 1*sσ*_*g*_ to 2*pσ*_*u*_ states to be


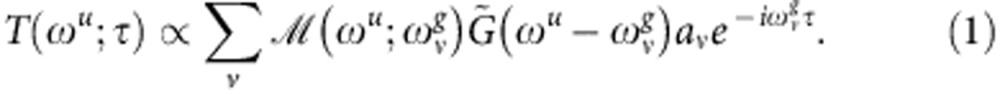


The derivation of this equation is presented in ref. 9. We also briefly describe the theoretical model used to obtain this equation in Supplementary Note 2.

On the right-hand side of equation (1), the wavepacket amplitude, *a*_*ν*_, is convolved with the positive-frequency part of the Fourier amplitude of the probe optical field (an optical field composed of the coherent superposition of the 3rd- and 5th-order harmonic fields in the actual experiment), 
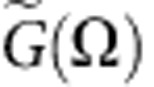
, with a delay-dependent phase factor, 
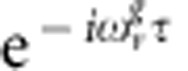
, and then mapped onto the repulsive state via the matrix elements of the electronic transition dipole moment, 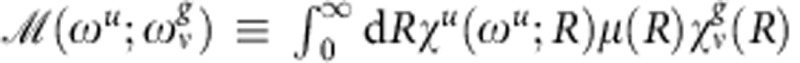. The nuclear wavefunction in the 2*pσ*_*u*_ state with an eigenenergy of *ħω*^*u*^ and the electronic transition dipole moment are defined as *χ*^*u*^(*ω*^*u*^;*R*) and *μ*(*R*), respectively.

Note that equation (1) is only valid under the condition that the probe pulse does not temporally overlap with the pump pulse (XUV APT) used for ionization. The magnitude square of *T*(*ω*^*u*^; *τ*) should be proportional to the delay-KER spectrogram of H^+^. As a result, we noticed that this spectrogram is very similar to that obtained when using the FROG technique to characterize the magnitude and phase of an ultrashort optical pulse. A detailed discussion of this similarity is given in ref. 9 and in Supplementary Note 2.

We have developed an iterative algorithm based on the generalized projection method in accordance with equation (1), which we call the matter-wave FROG (MW-FROG) algorithm hereafter. Note that we need a priori knowledge of the nuclear wavefunctions *χ*^*u*^(*ω*^*u*^;*R*) and 
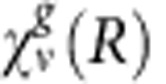
 and of the dipole moment *μ*(*R*) to specify 
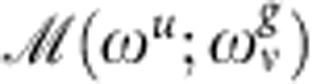
. Therefore, we used the adiabatic potentials of the 1*sσ*_*g*_ and 2*pσ*_*u*_ states obtained from theoretical calculations^15^ to determine *χ*^*u*^(*ω*^*u*^;*R*) and 
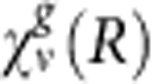
. The dipole moment is assumed to be a real number and proportional to *R*^16^.

### Application of MW-FROG

**Figure 3:**
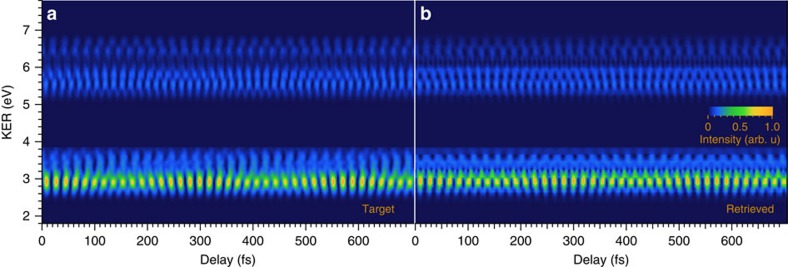
Implementation of MW-FROG (**a**) Target delay-KER spectrogram used in the MW-FROG algorithm. (**b**) Retrieved delay-KER spectrogram. The target spectrogram in (**a**) is obtained from the experimental data in Fig. 2a using the bandpass filter. Details of the MW-FROG algorithm are given in ref. 9.

Before implementing the MW-FROG algorithm, we need to adapt the measured spectrogram to obtain an appropriate target spectrogram. Because the phase information of the vibrational states is only contained in the beat frequency components 
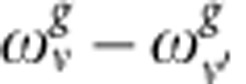
 (*ν*≠*ν*′), we apply a bandpass filter to the measured spectrogram to remove the frequency components irrelevant to the vibrational states, as shown in Fig. 2b. The magnitude square of the Fourier transform of the measured spectrogram clearly exhibits the beat frequencies with *ν*−*ν*′=1 and 2 as distinct peaks in this figure. The vibrational quantum numbers that we can specify from the experimental data are limited to <9, hence we restrict the range of *ν* to 0–8 in the MW-FROG algorithm. We extracted these frequency components in the areas marked with ellipses and then executed an inverse Fourier transform, resulting in the target spectrogram shown in Fig. 3a. The details of the data processing using the bandpass filter are explained in Supplementary Note 3 with Supplementary Figs 2,3 and 4.

**Figure 4:**
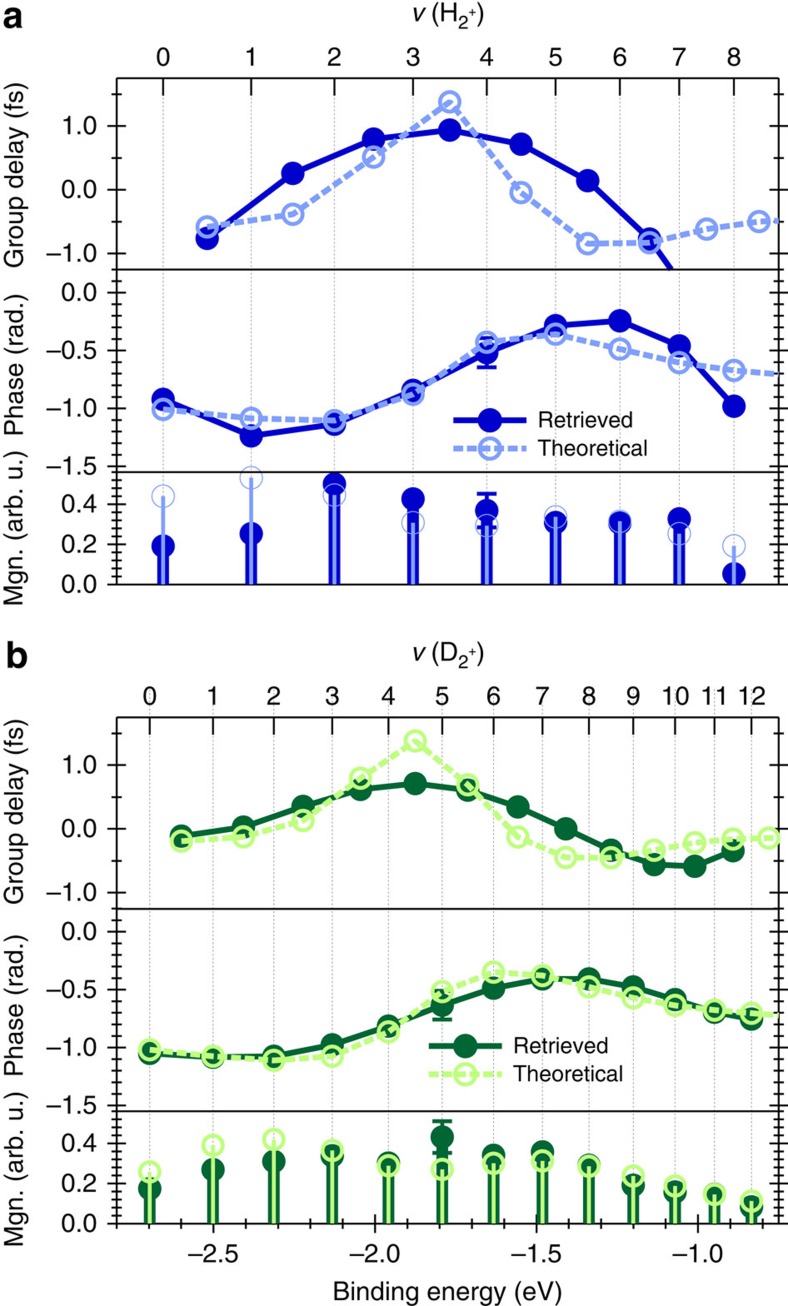
Comparison of *a*_μ_ obtained with MW-FROG and the theoretical model. Magnitude (bottom panel), phase (middle panel) and GD (top panel) of vibrational wavepacket amplitudes for 
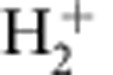
 (**a**) and 
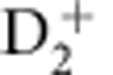
 (**b**). Retrieved quantities are depicted as solid circles, while the data obtained from the theoretical model discussed in the text are shown as hollow circles. Error bars in magnitude and phase indicate root-mean-square errors in the relevant vibrational numbers, which were evaluated by the numerical test for MW-FROG algorithm reported in ref. 9. The GD is defined as the finite difference between adjacent phases. Thus, we plot GDs at the midpoints between adjacent vibrational quantum numbers. The binding energy is measured from the dissociation limit of 
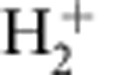
/
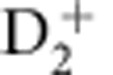
.

The delay-KER spectrogram retrieved from the target image in Fig. 3a is shown in Fig. 3b. The magnitude and phase of the retrieved *a*_*ν*_ are also shown as solid circles with bars and as solid circles with connected lines in the bottom and middle panels of Fig. 4a, respectively. We have addressed the retrieval of the gate field in Supplementary Note 4 and show the magnitude and phase of the retrieved gate field in Supplementary Fig. 5. We also describe the performance and accuracy of the MW-FROG algorithm in ref. 9. According to the analysis in ref. 9, the large error in the retrieved magnitude is due to the bandpass filter. The phase error is sufficiently small to detect phase modulation with a magnitude of 0.2 rad, in spite of the bandpass filtering, by sequentially optimizing the complex amplitude of *a*_*ν*_ and the polynomial expansion coefficients of the phase of *a*_*ν*_ in the MW-FROG algorithm. The details of the optimization process are described in ref. 9. A non-trivial modulation, which cannot be compensated by arbitrarily adjusting the group delay (GD) offset of a wavepacket, appears in the retrieved phase in the middle panel of Fig. 4a. A similar phase modulation can also be observed in the retrieved data for the vibrational wavepacket of 
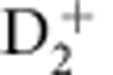
, as shown in the middle panel of Fig. 4b.

## Discussion

To clarify the origin of the phase modulation, we consider a theoretical model describing the transition amplitude of the ionization process^17^,^18^. In this model, the ionization process is described as a one-photon transition of a two-electron system, and the state of an ionized molecule is assumed to be a coherent superposition of the continuum electronic state accompanied by the 1*sσ*_*g*_ electronic state of a bounded electron in 
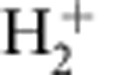
. The details of this model is shown in Supplementary Note 5 with a timing chart of the pump and probe pulses depicted as Supplementary Fig. 6. Then, we apply the approximation that the electronic transition dipole matrix is fixed to that at the equilibrium distance of H_2_. As a result, we can decompose the amplitude *a*_*ν*_ into the product of *a*_*ν*_^fc^ and *η*_*ν*_, *a*_*ν*_ ∝*a*_*ν*_^fc^*η*_*ν*_. The factor *η*_*ν*_ is composed of the sum of the contributions from the ionization process induced by the *n*th harmonic component, 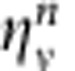, namely,


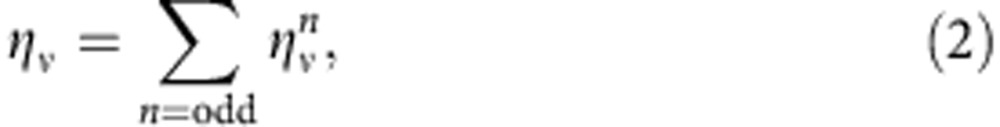


where we define 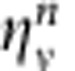 as


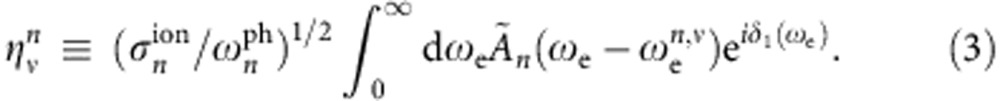


We denote the peak photon energy of the *n*th-order harmonic component as 
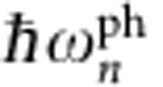
 and the ionization cross section on irradiation of the *n*th-order component as 
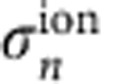
 in equation (3). The amplitude 
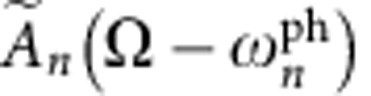
 is the Fourier amplitude of the *n*th-order harmonic component, the peak magnitude of which appears at 
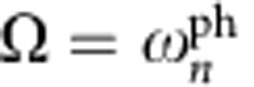
. The continuum energy of the electron generated with the peak-energy photon of the *n*th-order harmonic component is denoted as 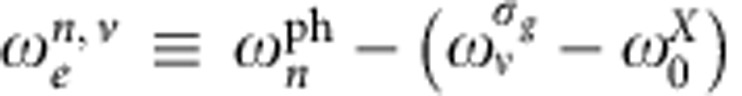 The phase shift in the asymptotic form of the Coulomb wavefunction^19^ of the ionized electron with an orbital angular momentum number of 1, *δ*_1_(*ω*_e_), is included in equation (3). We neglect the contribution to the ionization from the continuum states with other orbital angular momenta^18^. The derivation of equations (2) and (3) is described in Supplementary Note 5.

As we state in Supplementary Note 5, the phase shift *δ*_1_(*ω*_e_) does not exhibit a notable change in the continuum energy range of *ħω*_e_≳2 eV. Thus, we can recognize from equation (3) that the phase of 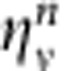 does not significantly differ with *ν* when the harmonic order *n* is ≥13 because the photon energies of these orders of the harmonic field are all more than 2 eV larger than the energy of the dissociation limit and the variable *ω*_e_ to be integrated in equation (3) always lies in this range. This result is consistent with the constant phase of *a*_*ν*_ obtained by assuming the conventional FC principle. We cannot expect, on the other hand, the same trend for the phase of 
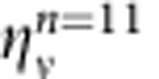
, where the photon energy 
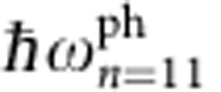
 is close to 
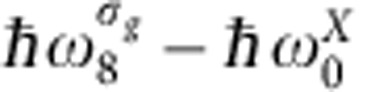
, owing to the rapid change in *δ*_1_(*ω*_e_) at *ω*_e_∼0.

**Figure 5:**
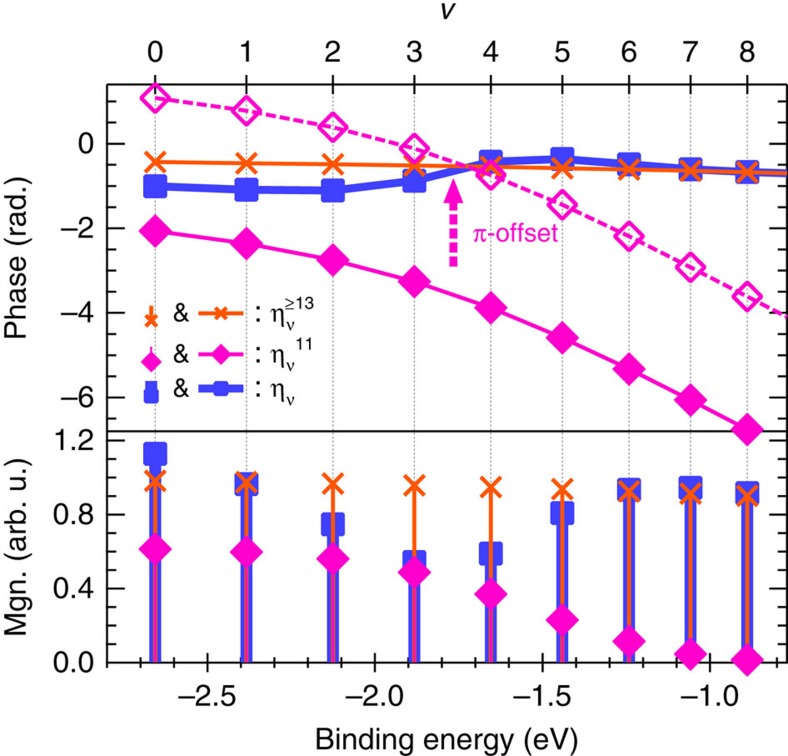
Magnitude (bottom panel) and phase (top panel) of *η*_*ν*_ for 
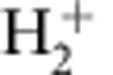
 obtained from our model calculation The contribution from H13 to H21 (
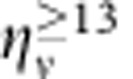
) and that only from H11 (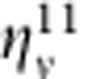) are shown as crosses and solid diamonds, respectively. Magnitude and phase of 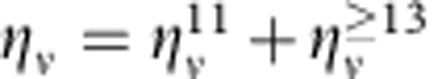 are depicted as solid squares in the bottom and top paneles, respectively. We also show the phase of 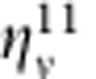 with the *π*-offset, depicted as hollow diamonds, to clearly show the phase difference of *π* between 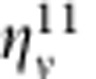 and 
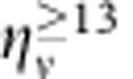
 at *ν*∼4.

The calculated results for 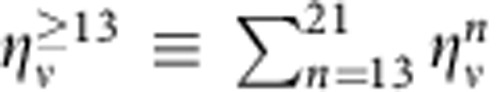 and 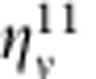 are depicted in Fig. 5. We approximated 
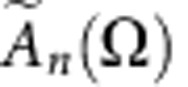
 as the square root of the Gaussian fit to the measured high-harmonic spectra with a group delay dispersion (GDD) of 0.7 × 10^−32^ s^2^ in this calculation. We explain how we estimated the GDD of the APT in Supplementary Note 5. The phases of *a*_*ν*_ calculated with four different GDDs are also depicted in Supplementary Fig. 7. We found that the phase of 
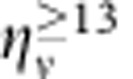
, depicted as crosses with connecting lines in the top panel of Fig. 5, decreases by only ∼0.2 rad when *ν* is increased from 0 to 8. The phase of 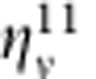, depicted as solid diamonds with connected lines, in contrast, decreases by more than 4 rad in the same range of vibrational quantum numbers. The significant feature of 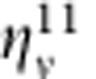 is that the phase *π*-shifted from that of 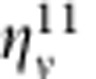 intersects the phase of 
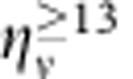
 in the region of 3<*ν*<4, shown as hollow diamonds connected by dotted lines in the top panel of Fig. 5. This leads to destructive interference when we compute 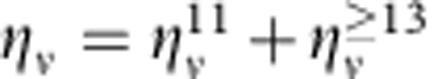.

We also show the calculated *η*_*ν*_ in Fig. 5. The dip of |*η*_*ν*_| around *ν*≃;3, shown as solid squares with bars in the bottom panel of Fig. 5, is evidence of destructive interference due to the phase difference of *π*. The phase of *η*_*ν*_, depicted as solid squares with connecting lines in the top panel of Fig. 5, is also modulated for the same reason. The resultant magnitude and position of the phase modulation are in good agreement with the experimental data, shown as hollow circles with connecting dashed lines in the middle panel of Fig. 4a. The good agreement between the retrieved and calculated phases in 
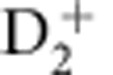
 is also exhibited in the middle panel of Fig. 4b. Hence, we conclude that we have observed a phase modulation of *a*_*ν*_ caused by the interference between the continuum electron wavefunction emerging with the absorption of the 11th-order harmonic component and that emerging with the absorption of the higher-order components.

The phase of a wavefunction appearing in an ionization process is closely related to the time delay required for ionization, which can be measured by the attosecond streaking technique^20^,^21^, two-colour high-order harmonic generation^22^, or from the electron interference spectrogram obtained by using two-colour above-threshold ionization^23^,^24^,^25^,^26^. The phase of *a*_*ν*_ in our study can also be interpreted as the time delay required for the formation of a vibrational wavepacket. We define the GD between the *ν*th and (*ν*+1)th vibrational states to be 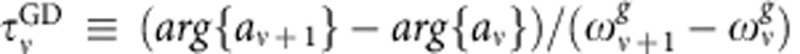, which should be a constant and exactly the same as the evolving time delay of the vibrational wavepacket if *arg*{*a*_*ν*_} linearly increases with the binding energy. The GDs of *a*_*ν*_ in 
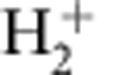
 obtained from the measured and modelled phases in our study both exhibit convex profiles in the region of 3<*ν*<4, as shown in the top panel of Fig. 4a. This characteristic is also revealed in the GDs of *a*_*ν*_ in 
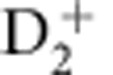
 in the region of 4<*ν* <5, as shown in the top panel of Fig. 4b. According to these figures, the 3rd and 4th vibrational states in 
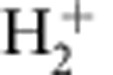
 (4th and 5th vibrational states in 
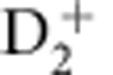
) are located ∼1 fs after the 1st and 2nd vibrational states in 
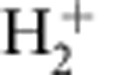
 (in 
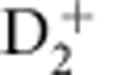
). Therefore, we can state that delay of ∼1 fs is required to settle the initial vibrational states of *ν*=0–8 (*ν*=0–12) on the 1*sσ*_*g*_ state of 
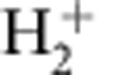
 (
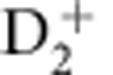
) through the ionization process from H_2_ (D_2_) owing to the interference of the continuum electron.

We note that the above consideration of the settling time is only meaningful in the delay range in which the pump and probe pulses do not temporarily overlap, because we assumed this to derive equations (1), (2) and (3). We have addressed this issue in Supplementary Note 6. A more accurate description to conclude this study may be the following: the delay-KER spectrogram obtained by irradiation of the probe pulse after a sufficient delay (≳15 fs) is consistent with the physical model having an intrinsic ∼1-fs settling time difference of the vibrational states at the sudden emergence of the initial wavepacket, even though the initial wavepacket is actually prepared after passing through a ∼5 fs APT.

In spite of this limitation, our study has revealed a new physical insight into the birth of a vibrational wavepacket with a time scale of 1 fs. In particular, it is very important that the interference of the wavefunction of a continuum electron perturbs the motion of the nuclei, and thus, we can expect the possible control of the initial state of general nuclear motion through a coherent process in ionization. While the ionization time in the liberation motion of an electron is still an important observation to be measured in the attosecond scientific field, the settling time of the coherent motion of a large quantum system initiated via the ultrafast ionization of an electronic system should also be a new area of research to be investigated to solve the fundamental problems in the quantum time response of matter.

## Additional information

**How to cite this article**: Nabekawa, Y. *et al*. Settling time of a vibrational wavepacket in ionization. *Nat. Commun.* 6:8197 doi: 10.1038/ncomms9197 (2015).

## Supplementary Material

Supplementary Information Supplementary Figures 1-7, Supplementary Notes 1-6 and Supplementary References

## Figures and Tables

**Figure:**
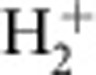

