# Supplementary material for: Settling time of a vibrational wavepacket in ionization
Source: Nat Commun. 2015 Sep 1;6:8197. doi: 10.1038/ncomms9197 (PMC4569855; doi:10.1038/ncomms9197)
Supplement: Supplementary Information — Supplementary Figures 1-7, Supplementary Notes 1-6 and Supplementary References [file ncomms9197-s1.pdf]

Supplementary Information

Supplementary Figures

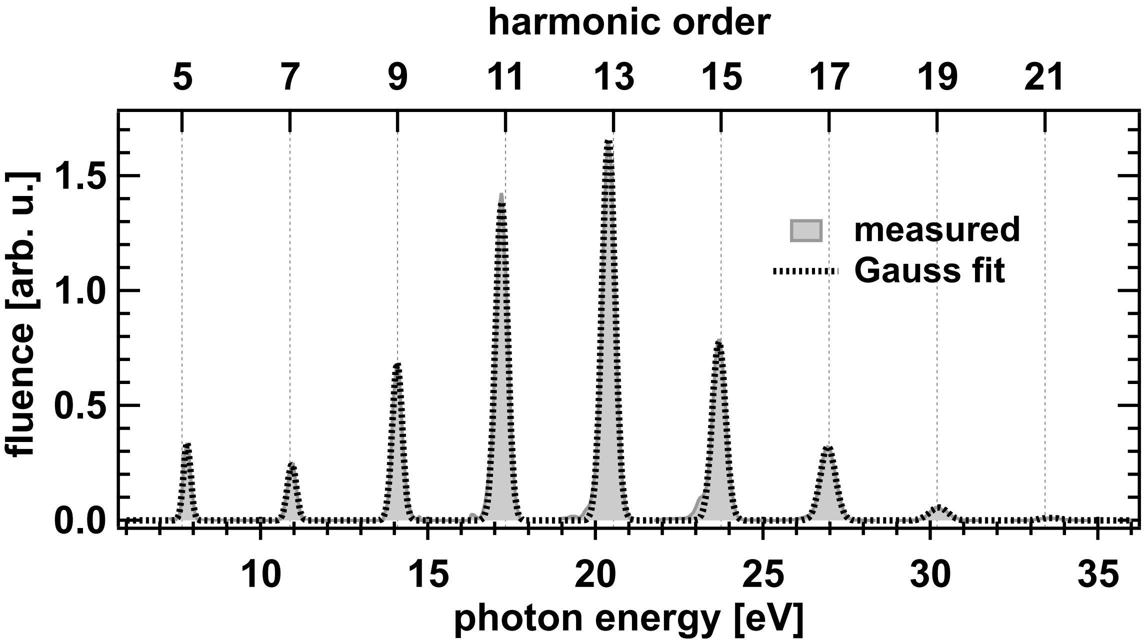

Supplementary Figure 1: Spectral shape of the APT at the focal point of the silicon carbide concave mirror estimated from the measured spectrum (shaded area). A Gaussian fit (dotted curve) of the measured profile is used to calculate the vibrational wavepacket amplitudes.

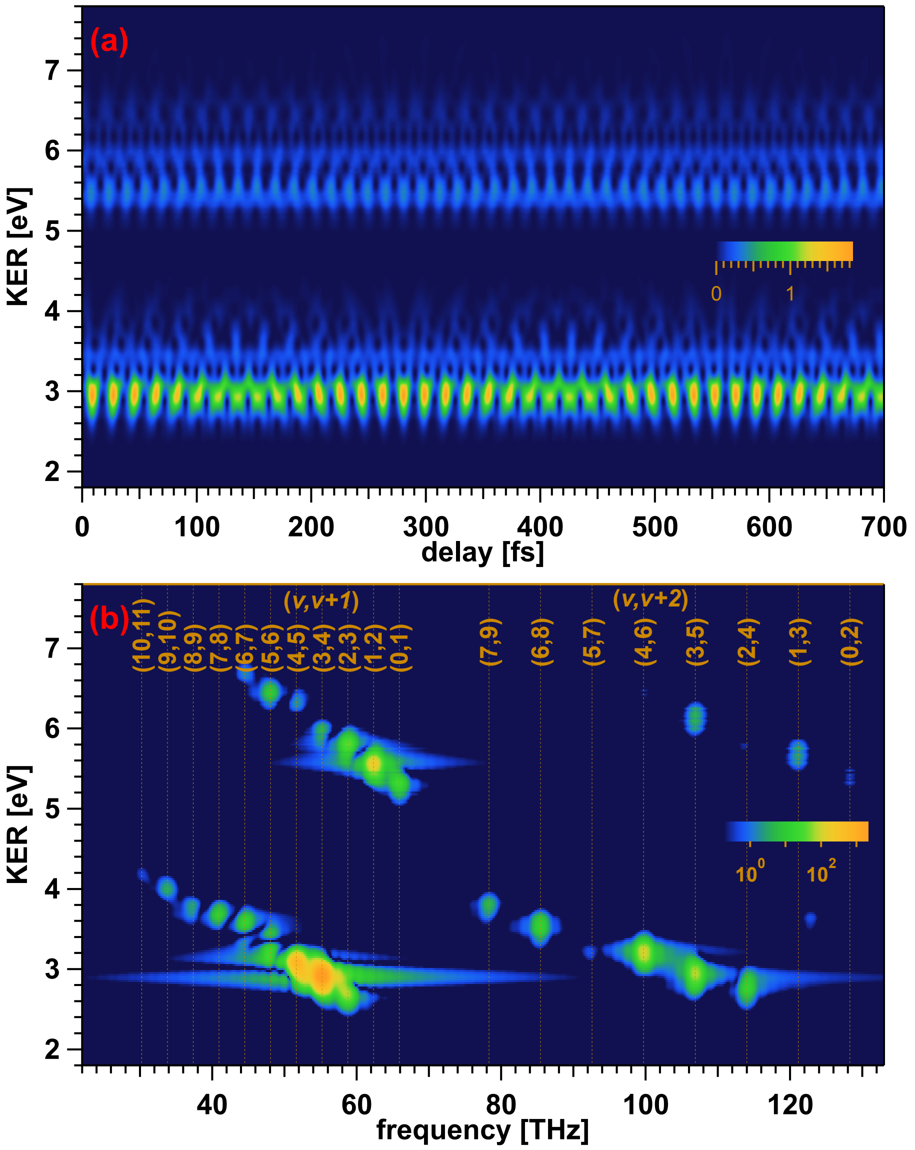

Supplementary Figure 2: (a) Delay-KER spectrogram calculated from $|T(\omega^u;\tau)|^2$ using Eq.(1) in the main text. (b) Magnitude square of the Fourier transform of the delay-KER spectrogram depicted in Supplementary Figure 2(a). The color scale used for the intensity is logarithmic.

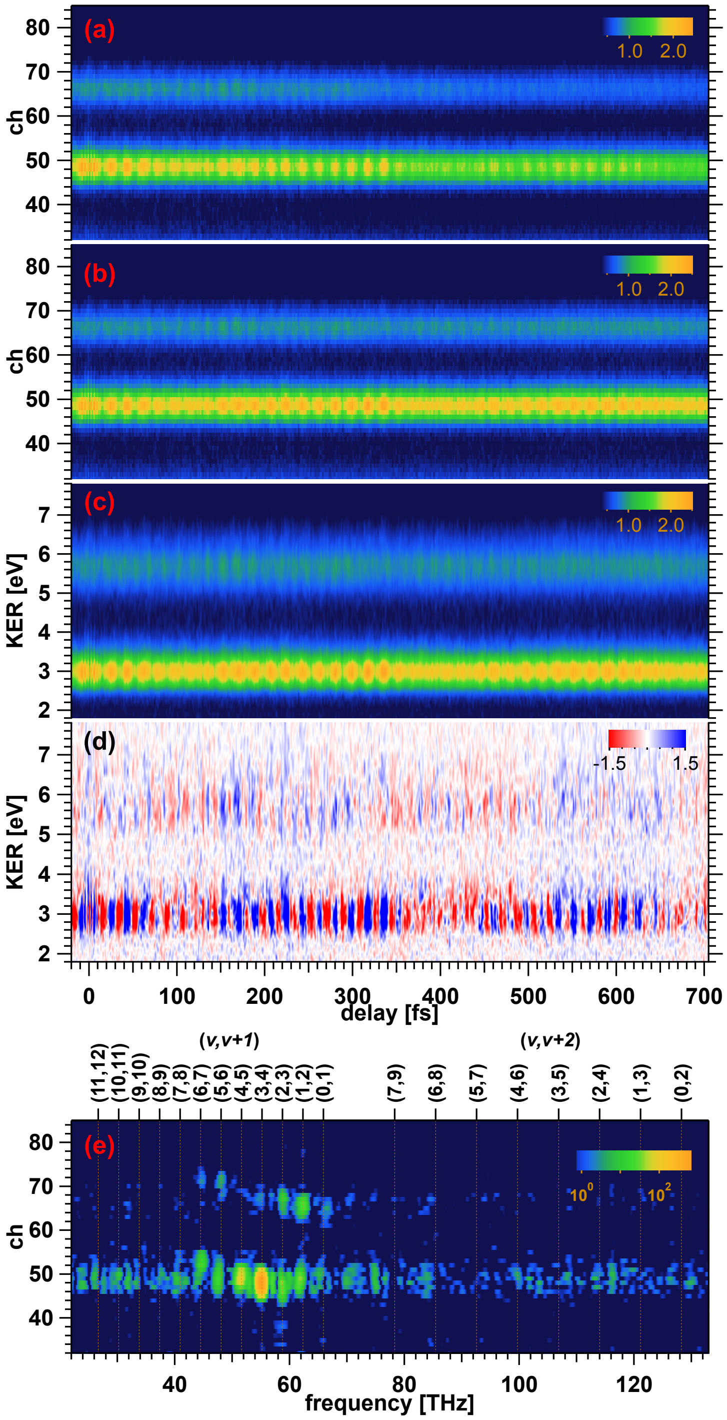

Supplementary Figure 3: Processing spectrogram of measured data. (a) Raw data with delay interpolation. The vertical axis indicates the channel number of the image sensor. (b) Spectrogram after compensation of the gradual decrease of the intensity against delay. We obtain the spectrogram shown in Supplementary Figure 3(e) by implementing the FT of this spectrogram. (c) Delay-KER spectrogram shown in Fig.2 in the main text. The vertical axis is converted from the channel to the KER by cubic spline interpolation. The KER is calibrated by comparing the channel positions of the beat frequency peaks appearing in Supplementary Figure 3(e) with the KER positions of the beat frequency peaks appearing in Supplementary Figure 2(b). (d) DC-subtracted image of the delay-KER spectrogram in Supplementary Figure 3(c). (e) Magnitude square of the FT of the spectrogram shown in Supplementary Figure 3(c). Pairs of vibrational numbers in parentheses are indicated on the top axis at the corresponding beat frequencies.

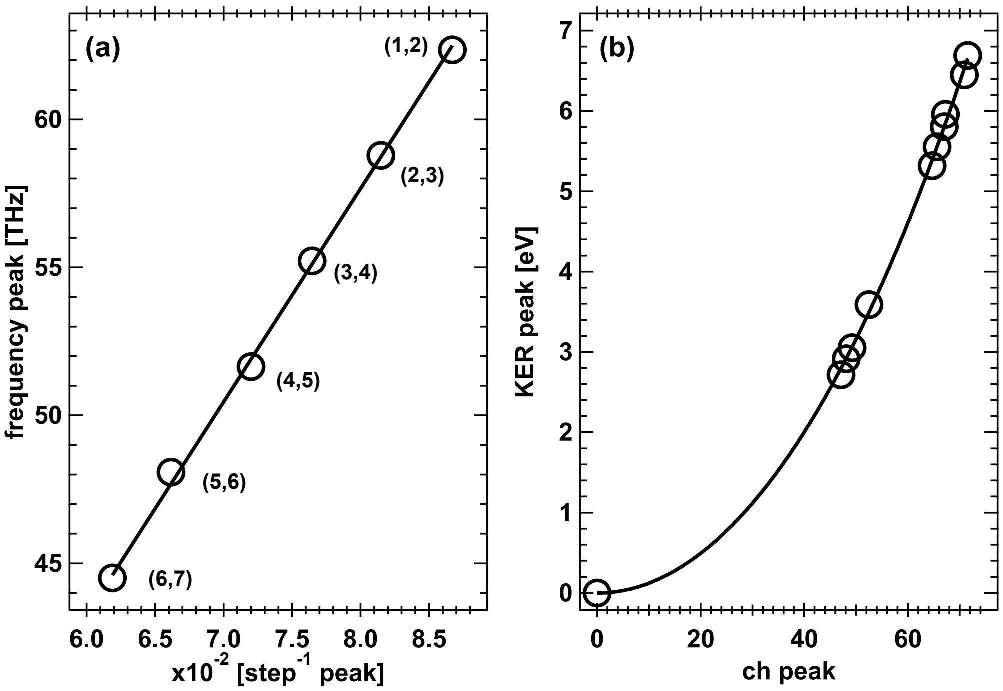

Supplementary Figure 4: Graphs exhibiting the calibration of the delay (a) and the KER (b) in the experimental spectrogram. (a) Correspondence between beat frequencies obtained from ref.[9] and the peak positions in the magnitude-squared FT spectrogram of the experimental data. Each pair of vibrational numbers between which a beat frequency is generated is depicted in parentheses near each plot. The constant of proportionality obtained by fitting a line to the plots is used to calibrate the delay. (b) KER peaks obtained from the calculated spectrogram in Supplementary Figure 2(b) plotted as circles against the peak channels in the magnitude-squared FT spectrogram of the experimental data, shown in Supplementary Figure 3(e).

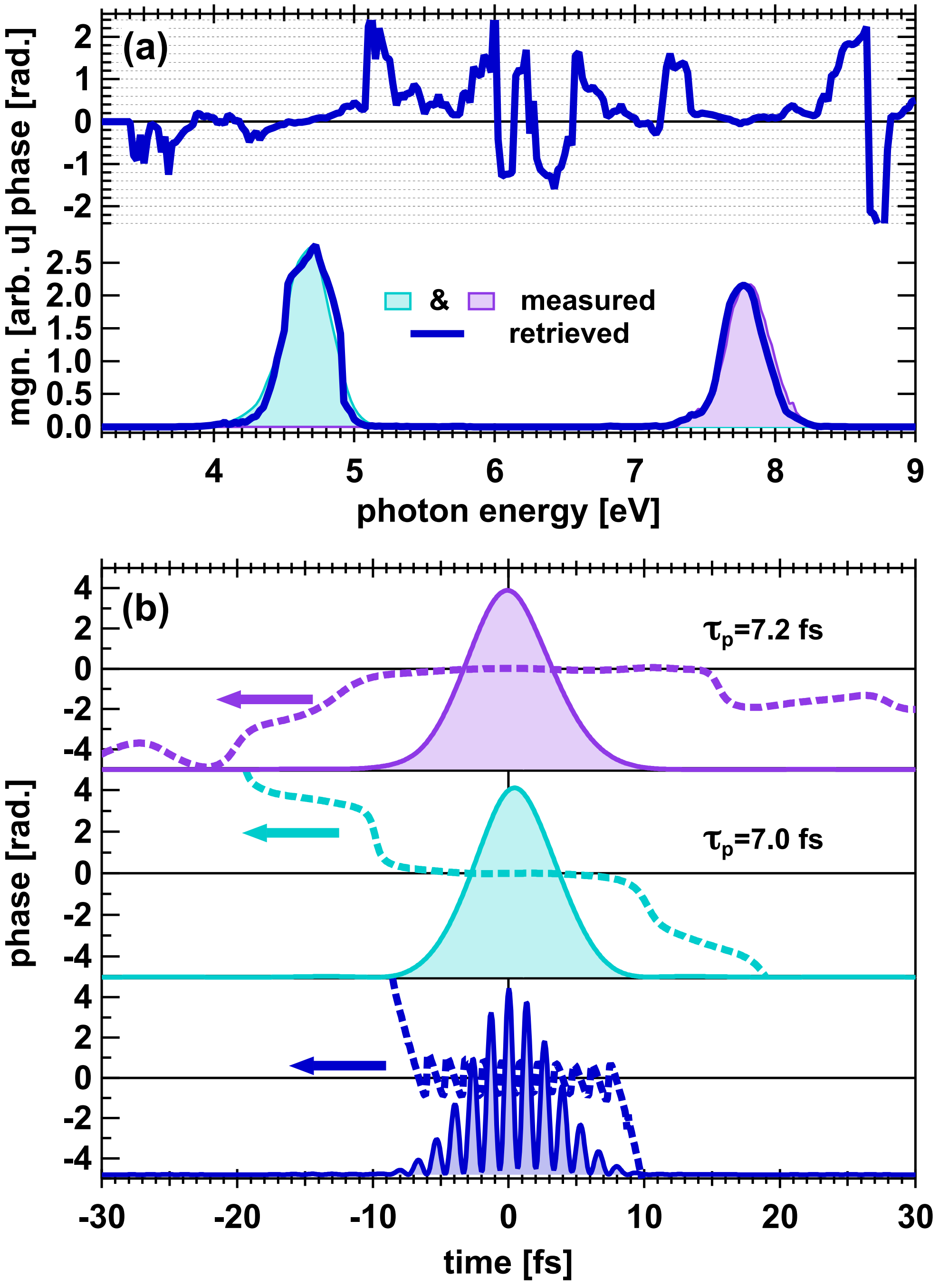

Supplementary Figure 5: (a) Magnitude (bottom panel) and phase (top panel) of the gate field retrieved from the experimental data. The spectral magnitudes of the 3rd- and 5th-order harmonic components separately measured with distinct spectrometers are depicted as shaded areas centered at ~4.7 eV and ~7.6 eV, respectively. (b) Shaded areas: temporal intensity profiles of the 5th-order (top panel), 3rd-order (middle panel) harmonic fields and the gate (bottom panel) field obtained by the synthesis of the two harmonic components. The dashed curve in each panel indicates the temporal phase in each pulse.

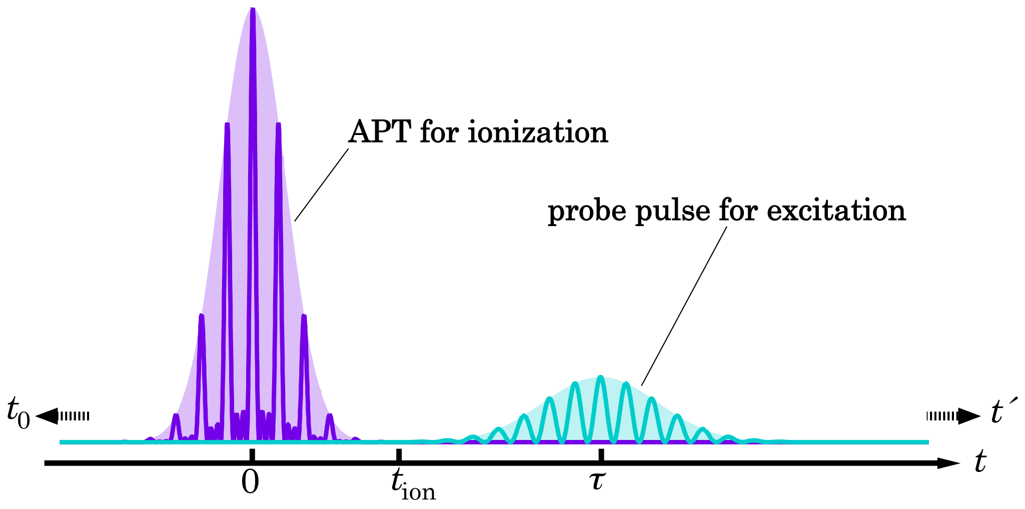

Supplementary Figure 6: Timing chart of the pump (ionization) and probe (excitation) pulses. We assume that the magnitude of the APT is sufficiently small at the end time of the pump pulse t_{ion}, while the delay time at the irradiation of the probe pulse (train) τ should be larger than t_{ion}.

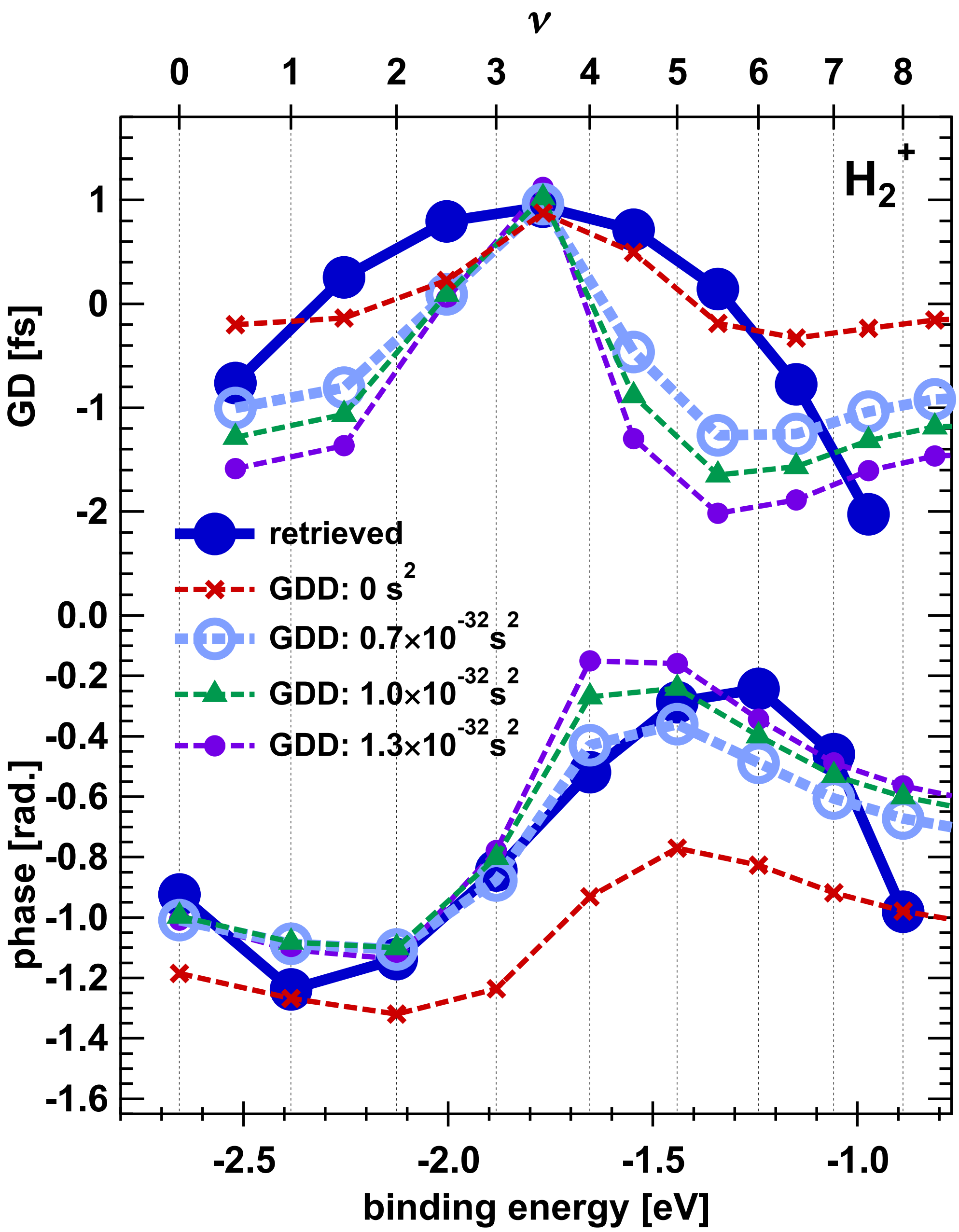

Supplementary Figure 7: Phase (bottom panel) and group delay (GD, top panel) of a_ν calculated using Eqs.(21), (24), and (26). The group delay dispersion (GDD) of the APT field is adjusted to 0 s^2 (crosses with dashed connecting lines), 0.7×10^{-32} s^2 (hollow circles with dashed connecting lines), 1.0×10^{-32} s^2 (triangles with dashed connecting lines), and 1.3×10^{-32} s^2 (solid circles with dashed connecting lines). The retrieved phase and GD are also depicted as solid circles with connecting lines.

Supplementary Notes

Supplementary Note 1: Experimental details

The design and performance of the titanium sapphire laser system, which delivers 14 fs pulses at a repetition rate of 100 Hz with a maximum energy of 40 mJ, were reported in ref.[1]. The laser pulse propagating from the grating pair compressor in a vacuum chamber is focused into a static gas cell filled with Xe gas to generate high-harmonic (HH) fields, forming an attosecond pulse train (APT). The condition for HH generation is the same as that reported in ref.[2].

After propagating for 4 m in the vacuum tube from the gas cell, thereby increasing the beam diameter to $\sim$3 mm, the APT is reflected from a pair of silicon harmonic separator mirrors to significantly reduce the energy of the fundamental laser pulse at the Brewster incident angle. The APT is spatially split into two replicas by the reflection near the boundaries of the silicon mirror pair in a parallel configuration. We can control the delay between the two replicas of the APT by adjusting the position of one of the silicon mirrors mounted on a piezo translation stage. The travel range of the piezo actuator is set to be 10-fold longer than that of the actuator used in our previous experiment to improve the frequency resolution by extending the delay scanning range from $\sim$100 fs[2] to $\sim$1 ps. After passing through an aperture with a diameter of 2 mm, which rejects long-trajectory components in the HH fields and decreases the energy of the remaining fundamental laser pulse, the APT pair is introduced into a vacuum chamber containing a concave mirror made of silicon carbide with a curvature of 200 mm and a velocity map imaging (VMI) spectrometer for ions.

The spectral profile of the APT at the focal point of the concave mirror, which is estimated from the spectrum measured using an XUV spectrograph and the efficiencies of the measuring apparatus, is shown as the shaded area in Supplementary Figure 1. The spectrum of the 3rd-order harmonic component in the APT is separately measured and shown in Supplementary Figure 5. The duration of each attosecond pulse in the APT is assumed to be 320 as[3] and the duration of the APT envelope is estimated to be approximately 5 fs from the measurement of the interferometric autocorrelation (IAC)[4, 5].

The fluence of the 5th-order harmonic is estimated to be 24 mJ/cm^2 from the measured pulse energy in front of the SiC concave mirror and the calculated focal spot size, resulting in a peak intensity of 3×10^{12} Wcm^{-2}. The intensity of the 3rd-order harmonic is similar to that of the 5th-order harmonic. The peak intensity of the 11th-order harmonic component is evaluated to be $\sim5\times10^{13}$ Wcm^{-2} by assuming the pulse width to be 5 fs. Thus, the peak intensity of the APT in the XUV region is higher than 10^{14} Wcm^{-2} owing to the coherent addition of multiple harmonic components.

The VMI ion spectrometer consists of three electrodes to accelerate ions and a microchannel plate (MCP) with a phosphor screen to measure the positions of ions. The fluorescence from the phosphor screen is relay-imaged onto a scientific CMOS sensor in a high-speed camera. We measure the angularly resolved momentum distribution as a fluorescent image on the phosphor screen by adjusting the voltages applied to the electrodes such that the velocity of ions is correctly mapped to the positions of the detected ions on the MCP. We apply a pulsed gate voltage with a duration of 100 ns to the MCP so as to discriminate the H^+ ions from other ions. Note that the background signal originating from the huge number of parent ions, which was a serious issue when analyzing the VMI data in the previous experiment[2], is completely removed by modification of the high-voltage pulser used for the MCP.

The target H_2 molecules are supplied in the form of a gas jet ejected from a piezo gas valve, which is attached to the back of the repeller electrode in the VMI spectrometer. A small bore with a diameter of 1 mm at the center of the repeller electrode skims the central part of the gas jet, while the density of the target molecules at the focal position of the APT is significantly higher than that of a molecular beam supplied from a differentially pumped chamber partitioned with a skimmer used in the previous experiment[2].

We note that the H^+ yield in the low kinetic energy release (KER) region (KER$\lesssim$1 eV) originating from the unfocused APT passing to the side of the gas jet is also much higher than that observed in the previous experiment[2]. This unwanted background H^+ yield makes it difficult to find the vibrational wavepacket motion of H_2^+, which should in principle appear in this low-KER region upon excitation with the fundamental laser pulse. Hence, we do not use the spectrum in this low-KER region for the reconstruction of the wavepacket.

We maintain the ambient pressure of the VMI chamber at $\sim10^{-4}$ Pa by setting the duration of the pulsed voltage applied to the piezo valve to as short as possible (20 μs) with a 100 Hz repetition rate. The backing pressure of the piezo valve is typically 1 atm, and thus the temperature of the target gas is expected to be much lower than room temperature.

The harmonic components of the 11th order and above contained in the APT contribute to the ionization of H_2 molecules, as shown in Fig.1 in the main text, while the 3rd- and 5th-order harmonic components in the APT excite H_2^+ molecular ions from the $1s\sigma_g$ to $2p\sigma_u$ states by one-photon absorption, resulting in dissociation. The pulse durations of the 3rd and 5th orders are measured to be 7.0 fs and 7.2 fs, shown as light blue and violet curves in Supplementary Figure 5(b) in this supplementary information, respectively. We clearly observed evidence of vibrational wavepacket motion by recording the KER of fragments, H^++H, by scanning the delay of the second probe pulse, as shown in Fig.2(a) in the main text.

During the delay-scanning measurement, a scientific CMOS camera is synchronously operated with the laser pulse at a repetition rate of 50 Hz. Hence, we acquire the two-shot accumulation of the VMI of the H^+ ions at every second shot of the laser pulse by setting an acquisition time of 20 ms. We have recorded 250 VMI data (during 500 laser shots), each with an increments of 790 nm of the piezo translation stage. We applied 530 increments for each scan of the delay and scanned the delay 6 times, resulting in the accumulation of data for 3000 laser shots at each delay point. We measured the harmonic spectrum, and then adjusted the laser system and the harmonic beam line before each scan of the delay so as to reproduce the ideal spectrum recorded at the time when we

measured the IAC trace of the APT.

Supplementary Note 2 : Physical model of the probe process

The electronic states relevant to the probe process are the ground state ($1s\sigma_g$) and the first excited state ($2p\sigma_u$). We may consider another probe process via the excited state of $2p\pi_u$ with one-photon absorption of the 9th-order or a higher-order harmonic component. In additional experiments performed after improving our high-order harmonic beam line to increase the APT intensity, we observed a significantly small contribution of this process to the KER spectrum of H^+/D^+ at $\sim$4.8 eV. We did not, however, observe such a contribution to the KER spectrum originating from the $2p\pi_u$ state in the delay-KER spectrogram shown in Fig.2(a) in the main text. Thus, we can neglect the contribution of the $2p\pi_u$ state, and we only consider a two-level system with eigenvectors of $|g\rangle=(0,1)^T$ for the ground electronic state of $1s\sigma_g$ and $|u\rangle=(1,0)^T$ for the excited electronic state of $2p\sigma_u$. The nuclear system in the ground/excited state evolves in accordance with the nuclear Hamiltonian $H_{g/u}(R)\equiv-\hbar^2/(2M)\cdot\partial^2/\partial R^2+V_{g/u}(R)$, where R and M are the nuclear distance and reduced hydrogen mass, respectively. The adiabatic potential in the ground/excited electronic state is notated as $V_{g/u}(R)$. Hence, we can describe the molecular Hamiltonian composed of the electronic states accompanying the nuclear degrees of freedom as $\widehat{H}_0(R)=H_g(R)\,|g\rangle\,\langle g|+H_u(R)\,|u\rangle\,\langle u|$. We assume that the vibrational wavepacket in the ground state is generated at time $t=0$ and that the probe pulse, which is composed of the coherent synthesis of the 3rd- and 5th-order harmonic components of a Ti:sapphire laser pulse, is irradiated at the delay time τ. We express the field amplitude of the probe pulse as $E(t-\tau)$. The transition between the ground and excited states is caused by a dipole of $\mu(R)$, hence the interaction Hamiltonian, denoted as $\widehat{V}(R;t)$, is expressed as $\widehat{V}(R;t)=\mu(R)E(t-\tau)\,|u\rangle\,\langle g|+\mu(R)E(t-\tau)\,|g\rangle\,\langle u|$ if we assume $E(t-\tau)$ to be a real-valued electric field.

The time evolution of a molecular state, $|\psi(R,t)\rangle$, is governed by the Schrödinger equation $i\hbar\partial/\partial t|\psi(R,t)\rangle=(\widehat{H}_0(R)+\widehat{V}(R,t))|\psi(R,t)\rangle$. The transition amplitude is the inner product of $|\psi(R,t)\rangle$, which is the solution of the Schrödinger equation under an appropriate initial condition, and the final state, $|\psi^{\text{fin}}(R,t)\rangle$, which expresses the state to be measured with the VMI spectrometer. To solve the Schrödinger equation, we adopt the standard procedure of time-dependent perturbation theory by assuming that the amplitude $E(t-\tau)$ is sufficiently low so that we can neglect the high-order multiphoton effect. Introducing the state vector in the interaction picture, $|\psi_{\text{int}}(R,t)\rangle=\mathrm{e}^{-\widehat{H}_0(R)t/i\hbar}|\psi(R,t)\rangle$, the Schrödinger equation is converted to the interaction picture as follows:

$$\frac{\partial}{\partial t}\,|\psi_{\text{int}}(R,t)\rangle=\frac{1}{i\hbar}\widehat{V}_{\text{int}}(R,t)\,|\psi_{\text{int}}(R,t)\rangle\,, \tag{1}$$

where the interaction Hamiltonian in the interaction picture, $\widehat{V}_{\text{int}}(R,t)$, is defined as $\mathrm{e}^{-\widehat{H}_0(R)t/i\hbar}\widehat{V}(R,t)\mathrm{e}^{\widehat{H}_0(R)t/i\hbar}$. By integrating both sides of this equation from the initial time 0 to t', then repeatedly substituting the expression for $|\psi_{\text{int}}(R,t)\rangle$, we have

$$\begin{aligned}&|\psi_{\text{int}}(R,t')\rangle\\&=|\psi_{\text{int}}(R,t=0)\rangle\\&\quad+\frac{1}{i\hbar}\int_0^{t'}dt_1\widehat{V}_{\text{int}}(R,t_1)\,|\psi_{\text{int}}(R,t=0)\rangle\\&\quad+\left(\frac{1}{i\hbar}\right)^2\int_0^{t'}dt_1\int_0^{t_1}dt_2\widehat{V}_{\text{int}}(R,t_1)\widehat{V}_{\text{int}}(R,t_2)\,|\psi_{\text{int}}(R,t=0)\rangle\\&\quad+\ldots.\end{aligned} \tag{2}$$

By multiplying by $\mathrm{e}^{\widehat{H}_0(R)t'/i\hbar}$ and using the relation $|\psi_{\text{int}}(R,t=0)\rangle=|\psi(R,t=0)\rangle$, we obtain the solution of $|\psi(R,t')\rangle$ within the first-order approximation as

$$\begin{aligned}&|\psi(R,t')\rangle\\&=\Big[\mathrm{e}^{\widehat{H}_0(R)t'/i\hbar}\\&\quad+\frac{1}{i\hbar}\int_0^{t'}dt_1\mathrm{e}^{\widehat{H}_0(R)(t'-t_1)/i\hbar}\widehat{V}(R,t_1))\mathrm{e}^{\widehat{H}_0(R)t_1/i\hbar}\Big]\,|\psi(R,t=0)\rangle\,.\end{aligned} \tag{3}$$

Since the molecular Hamiltonian is composed of the sum of projection operators that commute with each other, namely, $(|g\rangle\,\langle g|)^2=|g\rangle\,\langle g|$, $(|u\rangle\,\langle u|)^2=|u\rangle\,\langle u|$, and $[|g\rangle\,\langle g|\,,|u\rangle\,\langle u|]=0$, we can decompose $\mathrm{e}^{\widehat{H}_0(R)t/i\hbar}$ into the nuclear part and electronic part as $\mathrm{e}^{\widehat{H}_0(R)t/i\hbar}=\mathrm{e}^{H_g(R)t/i\hbar}\,|g\rangle\,\langle g|+\mathrm{e}^{H_u(R)t/i\hbar}\,|u\rangle\,\langle u|$. Substituting this equation into Eq.(2), $|\psi(R,t')\rangle$ becomes

$$\begin{aligned}&|\psi(R,t')\rangle\\&=\Big[\mathrm{e}^{H_u(R)t'/i\hbar}\,|u\rangle\,\langle u|+\mathrm{e}^{H_g(R)t'/i\hbar}\,|g\rangle\,\langle g|\\&\quad+\frac{1}{i\hbar}\int_0^{t'}dt_1\mathrm{e}^{H_u(R)(t'-t_1)/i\hbar}\mu(R)E(t_1-\tau))\mathrm{e}^{H_g(R)t_1/i\hbar}\,|u\rangle\,\langle g|\\&\quad+\frac{1}{i\hbar}\int_0^{t'}dt_1\mathrm{e}^{H_g(R)(t'-t_1)/i\hbar}\mu(R)E(t_1-\tau))\mathrm{e}^{H_u(R)t_1/i\hbar}\,|g\rangle\,\langle u|\Big]\\&\qquad|\psi(R,t=0)\rangle\,,\end{aligned} \tag{4}$$

thus giving the solution of the Schrödinger equation.

Next we determine the initial and final states. We assume the initial state to be the vibrational wavepacket in the ground state, expressed as

$$|\psi(R,t=0)\rangle=\sum_\nu a_\nu\chi_\nu^g(R)\,|g\rangle\,. \tag{5}$$

We define the νth vibrational eigenfunction of $H_g(R)$ as $\chi_\nu^g(R)$, which satisfies the eigenequation $H_g(R)\chi_\nu^g(R)=\hbar\omega_\nu^g\chi_\nu^g(R)$, where $\hbar\omega_\nu^g$ is the vibrational energy of the νth vibrational state. We experimentally measure the KER of the H^++H system. Thus, the final state at the time of detection, t', with an MCP should be proportional to $\chi^u(\omega^u;R)\,|u\rangle$, where $\chi^u(\omega^u;R)$ is the dissociative wavefunction satisfying $H_u(R)\chi^u(\omega^u;R)=\hbar\omega^u\chi^u(\omega^u;R)$ with a KER of $\hbar\omega^u$ for the dissociative state. The time evolution of the dissociative state from the time of probe irradiation to the time of detection should be expressed as the phase factor

$e^{-i\omega^u(t'-\tau)}$. Hence, we assume the final state to be $|\psi^{\rm fin}(R,t')\rangle = \chi^u(\omega^u;R)e^{-i\omega^u(t'-\tau)}|u\rangle$.

By substituting the initial state and evaluating the inner-product of $\langle\psi^{\rm fin}(R,t')|$ and $|\psi(R,t')\rangle$ in Eq.(4), we obtain the transition amplitude $\rho(\omega^u;\tau,t')=\int_0^\infty dR\,\langle\psi^{\rm fin}(R,t')|\psi(R,t')\rangle$ to be

$$\rho(\omega^u;\tau,t') = \frac{1}{i\hbar}\sum_v\int_0^\infty dR\chi^u(\omega^u;R)\mu(R)\chi_v^g(R) \cdot\int_0^{t'}dt_1E(t_1-\tau)a_v e^{i(\omega^u-\omega_v^g)t_1}e^{-i\omega^u\tau}. \quad (6)$$

We impose a condition to approximate Eq.(6). The delay, τ, for the probe field is sufficiently large so as to satisfy the condition of $E(-\tau)\simeq 0$ (or $T_p \le \tau$, where T_p is the pulse (train) duration of the probe field). The detection time, t', should be infinitely large compared with τ. Under this condition, the time integral in Eq.(6) is approximately proportional to the Fourier transform (FT) of $\widetilde{E}(\Omega)\equiv\int dtE(t)e^{i\Omega t}$. The transition amplitude, $T(\omega^u;\tau)$, giving by Eq.(1) in the main text, is proportional to the approximated $\rho(\omega^u;\tau,t'\to\infty)$ described as

$$T(\omega^u;\tau) \propto \sum_v \mathscr{M}(\omega^u;\omega_v^g)\widetilde{E}(\omega^u-\omega_v^g)a_v e^{-i\omega_v^g\tau}, \quad (7)$$

where $\mathscr{M}(\omega^u;\omega_v^g)$ is equal to $\int_0^\infty dR\chi^u(\omega^u;R)\mu(R)\chi_v^g(R)$, which is defined in the main text. The frequency difference $\omega^u-\omega_v^g$ in the argument of $\widetilde{E}$ is always positive, hence only the positive-frequency part of $\widetilde{E}(\omega^u-\omega_v^g)$ contributes to $T(\omega^u;\tau)$. Therefore, we can replace $\widetilde{E}(\omega^u-\omega_v^g)$ by $\widetilde{G}(\omega^u-\omega_v^g)$, which is the FT of the complex field amplitude of the probe (gate) field, giving Eq.(1) in the main text. The delay-KER spectrogram is obtained from $|T(\omega^u;\tau)|^2$.

We notice that the transition amplitude $T(\omega^u;\tau)$ is very similar to the correlation amplitude $S(\Omega;\tau)$, described as

$$S(\Omega;\tau) = \int dt\varepsilon(t)G(t-\tau)e^{i\Omega t} = \int d\omega\widetilde{G}(\Omega-\omega)\widetilde{\varepsilon}(\omega)e^{-i\omega\tau}, \quad (8)$$

which is used in the frequency-resolved optical gating (FROG) technique to characterize the ultrashort optical field $\varepsilon(t)$. The delay-frequency spectrogram used to apply the FROG technique is given by $|S(\Omega;\tau)|^2$. The physical quantity corresponding to the complex function $S(\Omega;\tau)$ is the amplitude of a classical optical electric field if the correlated optical field is generated via a nonlinear interaction, such as sum-frequency generation in a nonlinear crystal, between the classical optical electric fields $\widetilde{G}(\Omega)$ and $\widetilde{\varepsilon}(\omega)$.

The function $S(\Omega;\tau)$ may also be the probability amplitude of a transition between quantum states. The best-known example of this type of FROG technique is the 'complete reconstruction of attosecond bursts' (CRAB)[6] technique, in which $S(\Omega;\tau)$ is the transition amplitude to the continuum electronic state through the ionization process of an atom irradiated by an attosecond pulse and a near-infrared laser pulse with a scanning delay. Based on the formal correspondence between the gate function in the CRAB technique, which contains the phase factor of the classical action of an electron traveling in a near-infrared laser field, and the ordinary gate function in the conventional optical FROG technique, Mairesse and Quéré demonstrated the application of the FROG algorithm to the delay-kinetic energy spectrogram of an ionized electron[6]. The convergence of the retrieval for the two-dimensional spectrogram with a one-dimensional compact support was also confirmed in this study. We found a similar formal correspondence between Eq.(1) in the main text and Eq.(8), and thus, we conclude that we can apply the FROG algorithm to the delay-KER spectrogram of a fragment ion.

The applicability of the FROG algorithm to Eq.(1) in the main text is more clearly proved by considering the discretized form of the equations for software implementation. In the FROG algorithm, the frequencies Ω and ω and the delay τ are all discretized with the forms of $\Omega_n=\Omega_{\rm offset}+n\Delta\Omega$, $\omega_{n'}=\omega_{\rm offset}+n'\Delta\omega$, and $\tau_m=\tau_{\rm offset}+m\Delta\tau$, where n, n', and m are integers. The constants $\Omega_{\rm offset}$, $\omega_{\rm offset}$ ($\tau_{\rm offset}$), $\Delta\Omega$, and $\Delta\omega$ ($\Delta\tau$) are the offset and incremental frequencies (delays) for the discretization, respectively. The correlation amplitude should have a discretized matrix form for (n,m) expressed as

$$S_{nm} = \sum_{n'}\widetilde{G}_{nn'}\widetilde{\varepsilon}_{n'}e^{-i\omega_{n'}\tau_m}, \quad (9)$$

where $\widetilde{G}_{nn'}\equiv\widetilde{G}(\Omega_n-\omega_{n'})$ and $\widetilde{\varepsilon}_{n'}\equiv\widetilde{\varepsilon}(\omega_{n'})$. The discretized form of Eq.(1) in the main text may be written as

$$T_{nm} = \sum_v \widetilde{G}'_{nv}a_v e^{-i\omega_v^g\tau_m}, \quad (10)$$

with $\omega_n^u\equiv\omega_{\rm offset}^u+n\Delta\omega^u$ and

$$\widetilde{G}'_{nv} \equiv \mathscr{M}(\omega_n^u;\omega_v^g)\widetilde{G}(\omega_n^u-\omega_v^g). \quad (11)$$

It is ensured that the FROG algorithm can be applied to retrieve $\widetilde{G}'_{nv}$ and a_v because of the complete correspondence between the mathematical forms of S_{nm} and T_{nm} in Eqs.(9) and (10). The gate field is obtained by dividing the retrieved $\widetilde{G}'_{nv}$ by $\mathscr{M}(\omega_n^u;\omega_v^g)$ in principle, although we optimize the gate field itself in the actual MW-FROG algorithm as shown in ref. [7].

For the above-mentioned reason, we need to determine the nuclear wavefunctions $\chi^u(\omega^u;R)$ and $\chi_v^g(R)$, and the transition dipole $\mu(R)$, by using a theoretical model to calculate $\mathscr{M}(\omega^u;\omega_v^g)$. Therefore, the application of $T(\omega^u;\tau)$ giving by Eq.(1) to the experimental spectrogram always includes errors originating from the discrepancy between the modeled $\mathscr{M}(\omega^u;\omega_v^g)$ and the actual $\mathscr{M}(\omega^u;\omega_v^g)$ of the H_2^+ system to be measured. We note, however, that errors only arise in the magnitude of $T(\omega^u;\tau)$. The phase of a_v is not affected by this discrepancy. This is because we define $\mathscr{M}(\omega^u;\omega_v^g)$ as a real function using the real functions $\chi^u(\omega^u;R)$, $\chi_v^g(R)$, and $\mu(R)$. The magnitude of $\mu(R)$ assumed in our model is approximately 5% higher than that of $\mu(R)$ obtained from a more accurate calculation at a nuclear distance of $\sim$170 pm, where the transition amplitude is maximized for the absorption of the 3rd harmonic component. This deviation may cause a $\sim$5% overestimation of the magnitude of the delay-KER spectrogram in

the vicinity of 3 eV. It may also slightly distort the magnitude distribution for a_ν, which should be, however, negligibly small compared with the magnitude of the error for a_ν arising from the bandpass filter used to process the experimental data.

The phase information of a_ν is only contained in the peaks of the beat (difference) frequencies appearing in the FT of the delay-KER spectrogram. These peaks reveal the relative phase differences between the different a_νs. This fact ensures that the bandpass filter used to appropriately extract the beat frequency components should be effective in suppressing the noise in the spectrogram without changing the phase information of a_ν, even though the accuracy of retrieval of the magnitude is significantly degraded using the bandpass filter. We have performed the accurate extraction of the beat frequency components by carefully calibrating the delay and KER of the experimental spectrogram such that the peaks of the beat frequency components in the experimental spectrogram coincide with those of the model spectrogram depicted in Supplementary Figure 2(b). The details of the calibration process are described in Supplementary Note 3. As a result, we obtained the bandpass filter depicted in Fig.2(b) in the main text.

We note that the KER spectrum obtained from the 3rd-order harmonic component does not exhibit beat frequencies at (0,1) and (1,2) in Supplementary Figure 2(b), and thus we cannot retrieve the phases of a_0 and a_1 without information from the KER spectrum obtained from the 5th-order harmonic component exhibiting these frequency peaks. The common beat frequencies between these two KER spectra ensure the consistency of the phase retrieval from a_2 to a_8. Therefore, both KER spectra are necessary for retrieving the phase of a_ν for all ν ranging from 0 to 8.

We also note that the passband of the filter at the beat frequency components between the next adjacent vibrational state, $(\nu,\ \nu+2)$, may be redundant because the phase information of these beat frequencies can be obtained from the beat frequency components of $(\nu,\ \nu+1)$ and $(\nu+1,\ \nu+2)$. Thus, the missing frequency components in the $(\nu,\ \nu+2)$ region in the experimental spectrogram shown in Fig.2(b) in the main text do not make it difficult to retrieve the phase of a_ν, while they may degrade the accuracy of magnitude retrieval. We have also demonstrated in ref.[7] that the MW-FROG algorithm for the phase retrieval is robust against noise, a reduction in the resolution of the KER, and magnitude modulation of the gate field.

Supplementary Note 3: Data processing

Before processing the experimental data, we generated a reference spectrogram by calculating $|T(\omega^u;\tau)|^2$ using Eq.(1) in the main text, as shown in Supplementary Figure 2(a). The details of this model calculation are described in ref.[7].

The magnitude square of the FT of $|T(\omega^u;\tau)|^2$ is also shown in Supplementary Figure 2(b), which exhibits beat frequency components of $(\omega^g_{\nu+1}-\omega^g_\nu)/2\pi$ and $(\omega^g_{\nu+2}-\omega^g_\nu)/2\pi$, resolved in the direction of the KER axis. This frequency-KER spectrogram is used for calibrating experimental data as mentioned later.

We processed the image data of the H^+ fragment recorded with the sCMOS camera by counting the light spots on the sCMOS sensor brighter than a threshold intensity for each delay step. This resulted in one fragment image being obtained from 1500 recorded data (3000 laser shots) by performing the counting analysis for each delay. We converted the fragment image into an angle-resolved velocity distribution by a conventional image inversion method[8]. The angle-resolved velocity distribution was angularly integrated, then arranged in accordance with the delay, resulting in a two-dimensional spectrogram, in which one of the axes is proportional to the delay and the other is proportional to the velocity.

In order to calibrate the delay, we performed the FT of the spectrogram with respect to the delay step number, then calculated the magnitude square of the complex FT spectrogram. Note that we padded the delay steps with zeros to increase the number of delay steps before performing the FT to improve the resolution in the frequency domain. The resultant magnitude-squared FT spectrogram exhibited distinct peaks corresponding to beat frequencies. We determined the peak positions ($P_{\rm pk}$) in the unit of inverse stepnumber (step^{-1}) by Gaussian fitting of the peaks. The peak positions expressed in this unit should coincide with the known beat frequencies[9] ($F_{\rm beat}$) used to calculate the reference spectrogram and the magnitude square of its FT in Supplementary Figures 2(a) and (b). By fitting the line $F_{\rm beat} = gP_{\rm pk}$ to the beat frequencies, as shown in Supplementary Figure 4(a), the inverse of the proportional constant, g, gives us the delay per unit step of g^{-1} =1.3872±0.0012 [fs/step].

After calibrating the delay, we interpolated the midpoint data for the delay by the cubicspline fitting in this two-dimensional spectrogram to prevent aliasing of the FT in the MW-FROG algorithm. As a consequence, we obtain the spectrogram shown in Supplementary Figure 3(a) as raw data. The origin of the delay is determined by the central peak of the optical interference fringes of the fundamental frequency component[2, 10] in the region with KER lower than 1.8 eV.

We observe a gradual decrease of the intensity accompanied by an increase of the delay in Supplementary Figure 3(a). This is caused by the gradual degradation of the reflectivity of the silicon beam separator mirrors irradiated with intense extreme ultraviolet pulses during the measurement. We compensated for this irrelevant change of the intensity so as to maintain a straight baseline of the line profile for the delay at each vertical channel . This is implemented by (i) extracting the average line profile by 3rd-order polynomial fitting to the line profile of the raw data, then (ii) renormalizing the line profile of the raw data using the average line profile. The resultant spectrogram is shown in Supplementary Figure 3(b).

The vertical axis (ch) in Supplementary Figure 3(b) should be converted to the KER of the H^++H system. In order to calibrate the KER, we performed the FT of the spectrogram shown in Supplementary Figure 3(b). The magnitude square of the FT spectrogram is shown in Supplementary Figure 3(e). The peak KERs ($K_{\rm pk}$) obtained by the model calculation depicted in Supplementary Figure 2(b) are plotted as circles in Fig.4(b) against the peak channels ($C_{\rm pk}$) at the beat frequency components appearing in Supplementary Figure 3(e). By fitting a quadratic function of the channel with a small quar-

tic correction term, $K_{\rm pk}{=}aC_{\rm pk}^2+bC_{\rm pk}^4$, which is shown as a solid curve in Supplementary Figure 4(b), we estimate the coefficients a and b to be $1.232{\pm}0.034{\times}10^{-3}$[eV/ch^2] and $1.36{\pm}0.78{\times}10^{-8}$[eV/ch^4], respectively. The resultant channel resolutions at ∼3 eV and ∼5.6 eV are ∼120 meV/ch and ∼180 meV/ch, respectively, which are much larger than that of the gate field of 25 meV/ch. Hence, we interpolated the KER profile at each delay with a channel resolution of 25 meV/ch by cubic spline fitting. Although the intensity of the spectrogram should be corrected so as to conserve the KER spectral density in this conversion, we preserve the KER profile in the spectrogram to enhance the low intensity of the profile at ∼5.6 eV. The calibrated delay-KER spectrogram is shown in Fig.2(a) in the main text and in Supplementary Figure 3(c).

The delay-KER spectrogram in Supplementary Figure 3(c) includes the H^+ fragment components, which are independent of the delay. This DC background components originate from the simultaneous ionization and dissociation processes induced by the pump or probe pulse itself, which includes all the harmonic components ranging from the 3rd to 21st-order. Hence, we subtracted the DC component before applying the bandpass filter to the delay-KER spectrogram in Supplementary Figure 3(c), as shown in Supplementary Figure 3(d). This leads to the elimination of the background tail of the high-DC component in the beat frequency range.

We determined the transmission band of the bandpass filter, shown as contours in Fig.2(b) in the main text, by extracting the KER profile at each beat frequency in Supplementary Figure 2(b). The positions of the KER peaks in the bandpass filter are adjusted to coincide with those in Supplementary Figure 2(b). The KER width of the filter, which is fitted to a Gaussian profile, is empirically set to twice that in Supplementary Figure 2(b) in order to recover the KER resolution.

Regarding the frequency filter, we adopted the delta function for the delay step, $\Delta\tau$, and the finite number of points, N, given by $\sin((N+1)(\omega-\omega_{\rm peak})\Delta\tau/2)/\sin((\omega-\omega_{\rm peak})\Delta\tau/2)$. The actual N is set to twice the number of points to reduce the endpoint effects.

We note that the passband of the filter is in good agreement with the beat frequency components in Fig. 2(b) in the main text owing to the accurate calibrations of the delay and KER in the experimental spectrogram by using the spectrogram in Supplementary Figure 2(b).

The processed data after passing through the bandpass filter in the Fourier domain does not include the DC component, while the FROG algorithm requires the target image signal to be greater than or equal to zero. Hence we added the DC component as the delta function accompanying the averaged KER profile of the processed data for the KER axis, and then applied the inverse Fourier transform (IFT). We obtained the target delay-KER spectrogram depicted in Fig.3(a) in the main text by truncating negative values that accidentally appeared as a result of the numerical error in the IFT spectrogram.

Supplementary Note 4: Retrieval of gate field

We show the gate field retrieved from the experimental data of the delay-KER spectrogram (Fig.3(a) in the main text) in Supplementary Figure 5(a). The magnitude profile in the bottom panel of this figure is in good agreement with that of the 3rd-order harmonic component, which is measured with a UV spectrometer, in the vicinity of ∼4.7 eV, and with that of the 5th-order harmonic component, measured with another XUV spectrograph, in the vicinity of ∼7.6 eV.

We have estimated the dispersions of the retrieved phase in each harmonic component by fitting a second-order polynomial. As a result, the group delay (GD) of the 3rd-order harmonic component is ∼0.5 fs larger than that of the 5th-order harmonic component. The group delay dispersions (GDDs) of the 3rd- and 5th-order harmonic components are estimated to be $4{\times}10^{-31}$ s^2 and $2{\times}10^{-30}$ s^2, respectively. The resultant differences in the GD and GDDs are sufficiently small to conclude that the gate field pulse is nearly Fourier-limited, and this result is consistent with the fact that the 3rd- and 5th-order harmonic components are generated in the perturbative regime of the nonlinear process.

The temporal intensity profiles of the 5th- and 3rd-order harmonic fields calculated from the retrieved spectral amplitude with the truncation of the irrelevant harmonic component are shown in the top and middle panels of Supplementary Figure 5(b), respectively. Temporal phases, depicted as dashed curves in these panels, do not exhibit notable chirps, resulting in nearly Fourier-limited pulses with durations of 7.0 fs (3rd-order harmonic) and 7.2 fs (5th-order harmonic). The temporal intensity profile of the gate field is obtained by the IFT of the retrieved spectral amplitude. The resultant temporal profile is depicted as the shaded area in the bottom panel of Supplementary Figure 5(b). In this figure, we can observe a pulse train due to the synthesis of the 3rd- and 5th-order harmonic components. The sawtooth structure of the temporal phase, depicted as a dashed curve, is due to artifacts originating from the phase-unwrapping routine near zero magnitudes.

The timing offset of the pulse train in the train envelope, or equivalently, the relative phase between the 3rd- and 5th-order harmonic components, does not affect the retrieval of a_v, and hence the retrieved gate pulse train in the bottom panel of Supplementary Figure 5(b) may have an arbitrary timing offset in the train envelope. The reason for this is explained as follows. The gate field amplitude in the frequency domain, described as $\widetilde{G}(\omega^u-\omega^g_v)$, can be decomposed into the sum of the amplitudes of the 3rd- and 5th-order harmonic components, namely $\widetilde{G}^{(3)}(\omega^u-\omega^g_v)+\widetilde{G}^{(5)}(\omega^u-\omega^g_v)$, and thus the transition amplitude is written as $T(\omega^u;\tau){=}T^{(3)}(\omega^u;\tau){+}T^{(5)}(\omega^u;\tau)$, where we define $T^{(i)}(\omega^u;\tau){\equiv}\sum_v\mathscr{M}(\omega^u;\omega^g_v)\widetilde{G}^{(i)}(\omega^u-\omega^g_v)a_v e^{-i\omega^g_v\tau}$ $(i=3,5)$. The delay-KER spectrogram is obtained from the equation $|T(\omega^u;\tau)|^2{=}|T^{(3)}(\omega^u;\tau)|^2+|T^{(5)}(\omega^u;\tau)|^2{+}2\mathscr{R}\{T^{(3)*}(\omega^u;\tau)T^{(5)}(\omega^u;\tau)\}$. The last term of this equation disappears because there is no spectral magnitude overlap between $\widetilde{G}^{(3)}(\omega^u-\omega^g_v)$ and $\widetilde{G}^{(5)}(\omega^u-\omega^g_v)$. Therefore, the delay-KER spectrogram is invariant with respect to the multiplication of the phase factors,

such as $T^{(3)}(\omega^u;\tau)e^{i\phi_3}$ and $T^{(5)}(\omega^u;\tau)e^{i\phi_5}$, and we can safely apply our MW-FROG algorithm to the experimental data even in the unlikely event that the relative phase might be unstable. We notice from this analysis that the 3rd- and 5th-order harmonic components separately contribute to the retrieval of a_v, while we treat the gate field as the superposition of these two harmonic components in the MW-FROG algorithm to ensure the consistency of the relative phases between the beat frequency components commonly appearing in the two KER regions.

We note that the magnitude of the 5th-order harmonic component relative to that of the 3rd-order harmonic component is overestimated because we do not correct the KER profile when we convert the vertical axis from velocity (channel number of the sCMOS sensor) to KER. Hence, the train structure of the actual gate field may be somewhat buried. The accuracy of phase retrieval is not adversely affected by the overestimation of the magnitude.

Supplementary Note 5: Theoretical model for ionization

We can find many publications concerning the formulation of the ionization process of a H_2 molecule[11], [12], [13], [14, 15]. The common idea[14] is based on the description of a two-electron system in which the ground electronic state, composed of two bound-state electrons, transfers to the electronic state composed of one bound-state electron and one continuum-state electron generated by the interaction of an optical field under the Born-Oppenheimer approximation. We apply this idea to the time-dependent perturbation calculation to solve the Schrödinger equation $i\hbar\partial/\partial t|\Psi(R,t)\rangle = (\widehat{H}^{\text{mol}}(R) + \widehat{V}^{\text{XUV}}(t))|\Psi(R,t)\rangle$, where $|\Psi(R,t)\rangle$ and $\widehat{H}^{\text{mol}}(R)$ are a vector expressing the electronic and nuclear states of the H_2 molecule and the Hamiltonian governing the H_2 molecular system, respectively. We assume that the dipole interaction induces the ionization. Thus, the interaction Hamiltonian, $\widehat{V}^{\text{XUV}}(t)$, is set to $\widehat{\mu}E^{\text{XUV}}(t)$, where $E^{\text{XUV}}(t)$ is the XUV field of an APT that reaches the peak intensity at the time of 0. The dipole operator for the two-electron system, $\widehat{\mu}$, is represented in terms of the electron coordinates $(\boldsymbol{r}_1, \boldsymbol{r}_2)$ as $\langle \boldsymbol{r}'_1, \boldsymbol{r}'_2|\widehat{\mu}|\boldsymbol{r}_1, \boldsymbol{r}_2\rangle = \delta^3(\boldsymbol{r}'_1 - \boldsymbol{r}_1)\delta^3(\boldsymbol{r}'_2 - \boldsymbol{r}_2)\sum_{i=1,2} \text{q}_\text{e}\boldsymbol{\epsilon}\cdot\boldsymbol{r}_i$, where we denote the polarization vector of the XUV field and the charge of an electron as $\boldsymbol{\epsilon}$ and q_e, respectively. The form of the equation to be solved is the same as that described in Supplementary Note 2. Hence, the molecular state at time t_{ion}, i.e., the solution of the equation, exhibits the same form as that of Eq.(3) and is described by the following equation:

$$|\Psi(R,t_{\text{ion}})\rangle = \Big[e^{\widehat{H}^{\text{mol}}(R)(t_{\text{ion}}-t_0)/i\hbar} + \frac{1}{i\hbar}\int_{t_0}^{t_{\text{ion}}} dt_1 e^{\widehat{H}^{\text{mol}}(R)(t_{\text{ion}}-t_1)/i\hbar}\widehat{V}^{\text{XUV}}(t_1))e^{\widehat{H}^{\text{mol}}(R)(t_1-t_0)/i\hbar}\Big] |\Psi(R,t=t_0)\rangle, \tag{12}$$

where we set the initial time when the H_2 molecule is prepared to t_0 and the time after the irradiation of the XUV APT field is denoted as t_{ion}. The time t_{ion} should be larger than the duration of the APT envelope to ensure the completion of the interaction, while we assume that t_{ion} is smaller than the delay of the probe field, τ.

The initial state at time t_0, $|\Psi^{\text{init}}(R)\rangle = |\Psi(R,t=t_0)\rangle$, should be proportional to the ground electronic state of a neutral H_2 molecule accompanying the ground vibrational state defined as $|\Psi_0^{\text{ntl},X}(R)\rangle = \frac{\chi_0^X(R)}{R}|\Psi^X(R)\rangle$. Hence, we write

$$\langle \boldsymbol{r}_1,\boldsymbol{r}_2|\Psi^{\text{init}}(R)\rangle = \langle \boldsymbol{r}_1,\boldsymbol{r}_2|\Psi_0^{\text{ntl},X}(R)\rangle e^{-i\omega_0^X t_0} = \frac{\chi_0^X(R)}{R}\Psi^X(\boldsymbol{r}_1,\boldsymbol{r}_2;R)e^{-i\omega_0^X t_0}, \tag{13}$$

in the electronic coordinates $(\boldsymbol{r}_1, \boldsymbol{r}_2)$ representation. The phase factor, $e^{-i\omega_0^X t_0}$, describes the propagation of the state from 0 to t_0 (< 0).

In this equation, the wavefunction with two bounded electrons is assumed to express the ground state of H_2 $(X^1\Sigma_g^+)$, which is denoted as $\Psi^X(\boldsymbol{r}_1,\boldsymbol{r}_2;R)$. The vibrational state of the nuclei is also assumed to be the ground state, hence we add a subscript of 0 to the vibrational wavefunction, $\chi_0^X(R)$. We assume that $|\Psi_0^{\text{ntl},X}(R)\rangle$ is the eigenvector of the molecular Hamiltonian $\widehat{H}^{\text{mol}}(R)$ satisfying $\widehat{H}^{\text{mol}}(R)|\Psi_0^{\text{ntl},X}(R)\rangle = \hbar\omega_0^X|\Psi_0^{\text{ntl},X}(R)\rangle$, where the energy of the ground vibrational state is denoted as $\hbar\omega_0^X$.

The electronic state in the ionic molecule, which is generated by the irradiation of a pump XUV APT field, should be composed of the $1s\sigma_g$ state in the H_2^+ molecular ion and the continuum state of one electron. When we set the quantum number of the vibrational state in the $1s\sigma_g$ electronic state, the continuum energy, and the angular momentum number of the continuum state of one electron to be ν, $\hbar\omega_\text{e}$, and p, respectively, the quantum state of the H_2^+ molecular ion $|\Psi_{\nu,\omega_\text{e},p}^{\text{ion}}(R)\rangle$ may be expressed as

$$\langle \boldsymbol{r}_1,\boldsymbol{r}_2|\Psi_{\nu,\omega_\text{e},p}^{\text{ion}}(R)\rangle = \frac{\chi_\nu^g(R)}{R}\widehat{\mathscr{A}}\psi^g(\boldsymbol{r}_1;R)\phi_p(\omega_\text{e};\boldsymbol{r}_2;R) \tag{14}$$

in the electronic coordinates representation.

The right-hand side of Eq.(14) includes the νth vibrational wavefunction, $\chi_\nu^g(R)$ (which was defined in Supplementary Note 2), in the ground electronic state of H_2^+, the wavefunction of which is denoted as $\psi^g(\boldsymbol{r}_1;R)$, and the wavefunction of the continuum electronic state, $\phi_p(\omega_\text{e};\boldsymbol{r}_2;R)$, with a continuum energy of $\hbar\omega_\text{e}$ and an angular momentum number of p. The symbol $\widehat{\mathscr{A}}$ is an operator that antisymmetrizes the wavefunction for the commutation of two electrons. The molecular state $|\Psi_{\nu,\omega_\text{e},p}^{\text{ion}}(R)\rangle$ satisfies the eigenequation $\widehat{H}^{\text{mol}}(R)|\Psi_{\nu,\omega_\text{e},p}^{\text{ion}}(R)\rangle = (\hbar\omega_\text{e} + \hbar\omega_\nu^{\sigma_g})|\Psi_{\nu,\omega_\text{e},p}^{\text{ion}}(R)\rangle$, where $\omega_\nu^{\sigma_g}$ is the νth vibrational energy measured from $\hbar\omega_0^X$. Note that $\hbar\omega_\nu^{\sigma_g}$ differs from $\hbar\omega_\nu^g$ in Eq.(7), which is specified as the binding energy in Figs.4 and 5 in the main text, by the offset energy of the dissociation limit, namely $\hbar\omega_\nu^{\sigma_g} = \hbar\omega_\nu^g +$(dissociation limit).

The amplitude a_v for the vth vibrational state, included in Eq.(5), should be proportional to the transition amplitude from the initial state in the H_2 molecule to the vth vibrational state in the $1s\sigma_g$ electronic state in the H_2^+ molecule. We assume that the continuum electron wavefunction accompanied by the H_2^+ molecule, which is not explicitly shown in Eq.(5), inherits the spectral coherence of a pump XUV APT field. Thus, we assume that the continuum electronic states contribute to this transition amplitude by the coherent superposition of wavefunctions with different $\hbar\omega_{\mathrm{e}}$ and p, which is equivalent to a continuum wavepacket. With this assumption, we define the final state of H_2^+ as

$$|\Psi_v^{\mathrm{fin}}(R,t_{\mathrm{ion}})\rangle = \sum_p \int_0^\infty d\omega_{\mathrm{e}} \mathrm{e}^{-i(\omega_{\mathrm{e}}+\omega_v^{\sigma_g})t_{\mathrm{ion}}} |\Psi_{v,\omega_{\mathrm{e}},p}^{\mathrm{ion}}(R)\rangle . \quad (15)$$

The phase factor $\mathrm{e}^{-i(\omega_{\mathrm{e}}+\omega_v^{\sigma_g})t_{\mathrm{ion}}}$ is multiplied by $|\Psi_v^{\mathrm{fin}}(R,t_{\mathrm{ion}})\rangle$ to express the propagation from time 0 to t_{ion} on the right-hand side of this equation.

The transition amplitude from the initial state to the vth vibrational state, a_v, is obtained by substituting $|\Psi^{\mathrm{init}}(R)\rangle$ in Eq.(13) into Eq.(12) and evaluating the inner product of $\langle\Psi_v^{\mathrm{fin}}(R,t_{\mathrm{ion}})|$ and $|\Psi(R,t_{\mathrm{ion}})\rangle$, resulting in

$$\begin{aligned} a_v &\propto \int_0^\infty dR R^2 \langle\Psi_v^{\mathrm{fin}}(R,t_{\mathrm{ion}})|\Psi(R,t_{\mathrm{ion}})\rangle \\ &= \sum_p \int_0^\infty d\omega_{\mathrm{e}} S_{v,p}(\omega_{\mathrm{e}}), \end{aligned} \quad (16)$$

where we define $S_{v,p}(\omega_{\mathrm{e}})$ as

$$\begin{aligned} &S_{v,p}(\omega_{\mathrm{e}}) \\ &\equiv \frac{1}{i\hbar}\int_0^\infty dR R^2 \langle\Psi_{v,\omega_{\mathrm{e}},p}^{\mathrm{ion}}(R)|\widehat{\mu}|\Psi_0^{\mathrm{ntl},X}(R)\rangle \\ &\quad \times \int_{t_0}^{t_{\mathrm{ion}}} dt_1 E^{\mathrm{XUV}}(t_1)\mathrm{e}^{i(\omega_{\mathrm{e}}+\omega_v^{\sigma_g}-\omega_0^X)t_1}. \end{aligned} \quad (17)$$

It is natural to approximate t_0 as $-\infty$ because the H_2 molecular ensemble is prepared a long time before the irradiation of the pump pulse field in the actual experiment. We show the schematic of the timing chart of the pump and probe pulses in Supplementary Figure 6 to clarify the notations related to time used throughout the main text and suppllimentary information. We have already assumed that the time t_{ion} is larger than the pulse duration of the APT envelope. Therefore, we approximate the time integral in Eq.(17) as

$$\begin{aligned} &\int_{t_0}^{t_{\mathrm{ion}}} dt_1 E^{\mathrm{XUV}}(t_1)\mathrm{e}^{i(\omega_{\mathrm{e}}+\omega_v^{\sigma_g}-\omega_0^X)t_1} \\ &\simeq \int_{-\infty}^{\infty} dt_1 E^{\mathrm{XUV}}(t_1)\mathrm{e}^{i(\omega_{\mathrm{e}}+\omega_v^{\sigma_g}-\omega_0^X)t_1} = \widetilde{E}^{\mathrm{XUV}}(\omega_{\mathrm{e}}+\omega_v^{\sigma_g}-\omega_0^X), \end{aligned} \quad (18)$$

because of $\int_{t_{\mathrm{ion}}}^{\infty} dt_1 E^{\mathrm{XUV}}(t_1)\mathrm{e}^{i(\omega_{\mathrm{e}}+\omega_v^{\sigma_g}-\omega_0^X)t_1} \simeq 0$. We define the Fourier amplitude of the APT pump field at at frequency Ω as $\widetilde{E}^{\mathrm{XUV}}(\Omega)$ in this equation. The argument in $\widetilde{E}^{\mathrm{XUV}}$ in Eq.(18), $\omega_{\mathrm{e}}+\omega_v^{\sigma_g}-\omega_0^X$, is always positive, and we can neglect the contribution from the ngative frequency part of this Fourier amplitude. Thus, we replace $\widetilde{E}^{\mathrm{XUV}}(\omega_{\mathrm{e}}+\omega_v^{\sigma_g}-\omega_0^X)$ as $\widetilde{E}^{\mathrm{XUV}+}(\omega_{\mathrm{e}}+\omega_v^{\sigma_g}-\omega_0^X)$ to express the positive frequency part of the Fourier amplitude of the APT.

By using the explicit form of the electronic transition matrix element for the dipole moment included in the former integral with respect to R in Eq.(17), we obtain $S_{v,p}(\omega_{\mathrm{e}})$ as

$$\begin{aligned} &S_{v,p}(\omega_{\mathrm{e}}) \\ &= \frac{1}{i\hbar}\int_0^\infty dR \chi_v^g(R)\chi_0^X(R)\mathscr{M}_p^{\mathrm{e}}(\omega_{\mathrm{e}};R)\widetilde{E}^{\mathrm{XUV}+}(\omega_{\mathrm{e}}+\omega_v^{\sigma_g}-\omega_0^X), \end{aligned} \quad (19)$$

with the electronic transition moment $\mathscr{M}_p^{\mathrm{e}}(\omega_{\mathrm{e}};R)$ defined as

$$\begin{aligned} &\mathscr{M}_p^{\mathrm{e}}(\omega_{\mathrm{e}};R) \\ &= \int d^3\boldsymbol{r}_1 \int d^3\boldsymbol{r}_2 \widehat{\mathscr{A}}[\psi^{g^*}(\boldsymbol{r}_1;R)\phi_p^*(\omega_{\mathrm{e}};\boldsymbol{r}_2;R)] \\ &\quad \times \sum_{i=1,2}(\mathrm{q}_{\mathrm{e}}\boldsymbol{\epsilon}\cdot\boldsymbol{r}_i)\Psi^X(\boldsymbol{r}_1,\boldsymbol{r}_2;R). \end{aligned} \quad (20)$$

The most important feature in Eq.(19) is that $S_{v,p}(\omega_{\mathrm{e}})$ contains a phase factor $\mathrm{e}^{i\Delta_p(\omega_{\mathrm{e}})}$ originating from the phase factor, $\mathrm{e}^{-i\Delta_p(\omega_{\mathrm{e}})}$, included in the continuum wavefaunction $\phi_p^*(\omega_{\mathrm{e}};\boldsymbol{r}_2;R)$ under the incoming wave boundary condition[16, 17, 18]. We can expect some kind of interference effect with the superposition of the continuum electronic states because the phase shift, $\Delta_p(\omega_{\mathrm{e}})$, alters in accordance with ω_{e}.

We apply some approximations to Eqs.(16)−(20) to roughly evaluate a_v. First, we replace $\mathscr{M}_p^{\mathrm{e}}(\omega_{\mathrm{e}};R)$ in Eq.(19) with $\mathscr{M}_p^{\mathrm{e}}(\omega_{\mathrm{e}};R_{\mathrm{eq}})$, where R_{eq} is the equilibrium distance of H_2 nuclei in the $X^1\Sigma_g^+$ state. This approximation is based on the fact that the wavefunction of the ground vibrational state, $\chi_0^X(R)$, is localized around the equilibrium distance, and thus, $\mathscr{M}_p^{\mathrm{e}}(\omega_{\mathrm{e}};R)$ only contributes to the R-integral in the vicinity of R_{eq}. This approximation enables us to decompose the overlap integral from other factors. By using this approximation and Eqs.(16) and (19), we obtain a_v as

$$a_v \propto a_v^{\mathrm{fc}}\eta_v, \quad (21)$$

with a_v^{fc} and η_v defined as

$$a_v^{\mathrm{fc}} = \int_0^\infty dR \chi_v^g(R)\chi_0^X(R) \quad (22)$$

and

$$\eta_v \equiv \sum_p \int_0^\infty d\omega_{\mathrm{e}} \mathscr{M}_p^{\mathrm{e}}(\omega_{\mathrm{e}};R_{\mathrm{eq}})\widetilde{E}^{\mathrm{XUV}+}(\omega_{\mathrm{e}}+\omega_v^{\sigma_g}-\omega_0^X), \quad (23)$$

The magnitude profile of $\widetilde{E}^{\mathrm{XUV}+}(\Omega)$ is proportional to the square root of the spectral profile shown in Supplementary Figure 1, which is composed of distinct spectra of multiple harmonic components. Hence, we can write $\widetilde{E}^{\mathrm{XIV}+}(\Omega) = \sum_{n=\mathrm{odd}}\widetilde{A}_n(\Omega-\omega_n^{\mathrm{ph}})$, where $\widetilde{A}_n(\Omega-\omega_n^{\mathrm{ph}})$ is the complex Fourier amplitude of the nth-order harmonic component. The magnitude of $\widetilde{A}_n(\Omega-\omega_n^{\mathrm{ph}})$ should be maximized around $\Omega=\omega_n^{\mathrm{ph}}$, and the bandwidth, $\Delta\omega_n^{\mathrm{ph}}$, is smaller than the frequency separation between the adjacent harmonic components. In accordance with this comblike feature of the harmonic spectrum,

we decompose η_ν into the sum of η_ν^n, which is the contribution from the nth harmonic component to η_ν, as follows.

$$\eta_\nu = \sum_{n=\mathrm{odd}} \eta_\nu^n, \tag{24}$$

where we define η_ν^n as

$$\eta_\nu^n \equiv \sum_p \int_0^\infty d\omega_e \mathscr{M}_p^e(\omega_e; R_{eq}) \widetilde{A}_n(\omega_e - \{\omega_n^{ph} - (\omega_\nu^{\sigma_g} - \omega_0^X)\}). \tag{25}$$

In order to evaluate the integral in Eq.(25), we approximate the phase shift, $\Delta_p(\omega_e)$, included in $\mathscr{M}_p^e(\omega_e; R_{eq})$ as that appearing in the asymptotic form of the Coulomb wavefunction, $\delta_\ell(\omega_e)$, for simplicity. The partial wave contributions other than the orbital angular momentum of $\ell = 1$ are also neglected[13, 15, 17], and hence, we substitute $\delta_1(\omega_e) = arg\{\Gamma(2 - i/k(\omega_e)z_B)\}$ for $\Delta_p(\omega_e)$ and omit the summation of p, where $k(\omega_e)$ is the wavenumber of the continuum electron and z_B is the Bohr radius. The phase shift $\delta_1(\omega_e)$ rapidly changes in the region of $\hbar\omega_e \lesssim 0.5$ eV. $\delta_1(\omega_e)$ at $\hbar\omega_e$=0.1 eV, for example, increases by more than 10 rad upon increasing $\hbar\omega_e$ to 0.3 eV. In contrast, the increase in the phase shift is only $\sim$1.2 rad when the energy is increased from 2 eV to 9 eV. We notice from Fig.1 in the main text that the continuum electron ionized with the 11th-order harmonic component should be in the low-energy region accompanying the rapid change in the phase shift in the bandwidth of the 11th-order harmonic component, while the continuum electron ionized with the other higher order harmonic components does not exhibit a significant change in the phase shift in the bandwidth of each harmonic component.

We do not expect such high contrast concerning the harmonic order for the phase terms depending on the electronic coordinates to be integrated on the right-hand side in Eq.(25). Therfore, we neglect these terms and approximate $\mathscr{M}_1^e(\omega_e; R_{eq})$ as $|\mathscr{M}_1^e(\omega_e^{n,\nu}; R_{eq})| e^{i\delta_1(\omega_e)}$, where $\omega_e^{n,\nu} \equiv \omega_n^{ph} - (\omega_\nu^{\sigma_g} - \omega_0^X)$ is the continuum energy of the electron generated with the peak energy photon of the nth-order harmonic component. The substitution of $|\mathscr{M}_1^e(\omega_e^{n,\nu}; R_{eq})|$ for $|\mathscr{M}_1^e(\omega_e; R_{eq})|$ is based on the fact that the contribution of $|\mathscr{M}_1^e(\omega_e; R_{eq})|$ to the integral with respect to ω_e in Eq.(25) mainly originates from the region where ω_e is around $\omega_e^{n,\nu}$ due to the relatively narrow bandwidth ($\sim$1 eV) of $\widetilde{A}_n(\omega_e - \omega_e^{n,\nu})$. As a result, we remove $|\mathscr{M}_1^e(\omega_e^{n,\nu}; R_{eq})|$ from the integral with respect to ω_e in Eq.(25).

Since the ionization crosssection at a photon energy of ω_n^{ph} is proportional to $\omega_n^{ph} |\mathscr{M}_1^e(\omega_e^{n,\nu}; R_{eq})|^2$[19], we replace $|\mathscr{M}_1^e(\omega_e^{n,\nu}; R_{eq})|$ by $(\sigma_n^{ion}/\omega_n^{ph})^{1/2}$, where σ_n^{ion} is the partial crosssection of the photoionization[20] to yield H_2^+ for the photon energy of $\hbar\omega_n^{ph}$. The vibrational state is not resolved by this substitution. The resultant factor η_ν^n is obtained by the equation

$$\eta_\nu^n \simeq (\sigma_n^{ion}/\omega_n^{ph})^{1/2} \int_0^\infty d\omega_e \widetilde{A}_n(\omega_e - \omega_e^{n,\nu}) e^{i\delta_1(\omega_e)}, \tag{26}$$

which is equivalent to Eq.(3) in the main text. The factor η_ν^n expresses the contribution of the nth-order harmonic component to the amplitude of the νth vibrational state.

We numerically calculated $\delta_1(\omega_e)$ and obtained η_ν using the ionization crosssection reported in ref.[20]. The phase shift diverges as ω_e decreases to 0, hence we restricted the lower bound for the ω_e-integral to a small positive quantity ($\sim$10μeV in energy unit) and confirmed that a small change in the lower bound does not significantly alter the result.

We estimated the group delay dispersion (GDD) of $\widetilde{E}^{XUV+}(\Omega)$ to be 1.3×10^{-32} s^2 in a past measurement using the mode-resolved autocorrelation technique[21]. We did not measure the exact GDD of $\widetilde{E}^{XUV+}(\Omega)$ in the present study, but we expect it to be similar to that measured in the past. We examined the alteration of the phase modulation of η_ν by setting the GDD to 0, 0.7×10^{-32}, 1.0×10^{-32}, and 1.3×10^{-32} s^2, as shown in Supplementary Figure 7. As a result, we found that the modulation depth of the phase slightly increases with the GDD, while the position of the modulation is fixed in the range of $3 < \nu < 4$. Therefore, we adopted a GDD of 0.7×10^{-32} s^2 in the model calculation because this value is in reasonably good agreement with the past measured data, which includes the positive material GDD of a tin filter with a somewhat inaccurate thickness of 0.1 μm.

Supplementary Note 6: Limitations of models and approximations

Our models for the pump and probe processes, described in Supplementary Notes 5 and 2, respectively, do not completely describe the actual processes in our experiment. In order to obtain the transition amplitude of the probe process in Eq.(1) in the main text (and Eq.(7) in Supplementary Note 2), we impose the initial value problem to the time-dependent Schrödinger equation and set the initial state to that given by Eq.(5), which should suddenly emerge at the time of 0. This model is correct only if the probe pulse is applied sufficiently after passing through the pump APT. In addition, the ionization occurs within a time scale much shorter than the shortest beat period of the vibration (15 fs), and the duration of the APT envelope in the pump field must be sufficiently short for the end time of the time integral in Eq.(17), t_{ion}, to be much less than 15 fs to apply the approximation used to obtain Eq.(19). Thus, the upper limit of the duration of the APT envelope is $\sim$7 fs, which approximately coincides with the duration of the Gaussian pulse formed with the frequency width of the highest beat frequency (65.925 THz) between the 0th and 1st vibrational states.

We stated in Supplementary Note 1 that the APT envelope duration of the pump field was estimated to be $\sim$5 fs from the IAC trace[4, 5] in a separate measurement. Even though the duration of the APT envelope used in this study might be somewhat different from that measured by the IAC due to small differences in the actual experimental conditions, the deviation of the APT field should be sufficiently small to satisfy the approximation condition mentioned above owing to the fact that we kept the ideal harmonic spectrum to reproduce a $\sim$5 fs APT envelope during the data acquisition performed by delay-scanning measurements in this study, as also stated in Supplementary Note 1.

Supplementary References

[1] Nabekawa, Y., Eilanlou, A. A., Furukawa, Y., Ishikawa, K. L., Takahashi, H., & Midorikawa, K., Multi-terawatt laser system generating 12-fs pulses at 100 Hz repetition rate. *Appl. Phys. B* **101**, 523-534 (2010).

[2] Furukawa Y., Nabekawa Y., Okino T., Eilanlou A. A., Takahashi E. J., Lan P., Ishikawa K. L., Sato T., Yamanouchi K., Midorikawa K. Resolving vibrational wave-packet dynamics of D_2^+ using multicolor probe pulses. *Opt. Lett.* **37**, 2922–2924 (2012).

[3] Nabekawa Y., Shimizu T., Okino T., Furusawa K., Hasegawa H., Yamanouchi K., Midorikawa K. Interferometric Autocorrelation of an Attosecond Pulse Train in the Single-Cycle Regime. *Phys. Rev. Lett.* **97**, 153904 (2006).

[4] Okino, T., Furukawa, Y., Eilanlou, A. A., Nabekawa, Y., Takahashi, E. J., Yamanouchi, K., & Midorikawa, K., Dissociative Ionization Dynamics of Nitrogen Molecule with Interferometric Autocorrelation of a-few-pulse Attosecond Pulse Train. *OSA Technical Digest, CLEO: QELS Fundamental Science (CLEO_QELS)* (OSA, 2013) QF2C.6.

[5] Okino, T., Furukawa, Y., Eilanlou, A. A., Nabekawa, Y., Takahashi, E. J., Yamanouchi, K., & Midorikawa, K., Attosecond Frequency Resolved Momentum Imaging of Two-photon Dissociative Ionization Dynamics of Nitrogen Molecule. *OSA Technical Digest, International Conference on Ultrafast Phenomena (UP)* (OSA, 2014) 09.Wed.P3.4.

[6] Mairesse, Y., & Quéré, F., Frequency-resolved optical gating for complete reconstruction of attosecond bursts. *Phys. Rev. A* **71**, 011401(R) (2005).

[7] Nabekawa Y., Furukawa Y., Okino T., Eilanlou A. A., Takahashi E. J., Yamanouchi K., Midorikawa K. Frequency-resolved optical gating technique retrieving the amplitude of a vibrational wavepacket. *Sci. Rep.* **5**, 11366 (2015), doi: 10.1038/srep11366.

[8] Vrakking, M. J. J., An iterative procedure for the inversion of two-dimensional ion photoelectron imaging experiments. *Rev. Sci. Instrum.* **72**, 4084-4089 (2001).

[9] Karr, J. P., & Hilico, L., High accuracy results for the energy levels of the molecular ions H_2^+, D_2^+ and HD^+, up to J = 2. *J. Phys. B* **39**, 2095-2105 (2006).

[10] Furukawa Y., Nabekawa Y., Okino T., Saugout S., Yamanouchi K., Midorikawa K. Nonlinear Fourier-transform spectroscopy of D_2 using high-order harmonic radiation. *Phys. Rev. A* **82**, 013421 (2010).

[11] Jiang Y. H., Rudenko A., Pérez-Torres J. F., Herrwerth O., Foucar L., Kurka M., Kühnel K. U., Toppin M., Plésiat E., Morales F., Martín F., Lezius M., Kling M. F., Jahnke T., Dörner R., Sanz-Vicario J. L., van Tilborg J., Belkacem A., Schulz M., Ueda K., Zouros T. J. M., Düsterer S., Treusch R., Schröter C. D., Moshammer R., Ullrich J. Investigating two-photon double ionization of D_2 by XUV-pump–XUV-probe experiments. *Phys. Rev. A* **81**, 051402(R) (2010).

[12] O'Neil S. V., Reinhardt W. P. Photoionization of molecular hydrogen. *J. Chem. Phys.* **69**, 2126–2142 (1978).

[13] Itikawa Y., Takagi H., Nakamura H., Sato H. Theoretical studies of photoionization of hydrogen molecules. *Phys. Rev. A* **27**, 1319–1327 (1983).

[14] Flannery, M. R., & Öpik, U., The photoionization of the hydrogen molecule from the ground electronic and vibrational state. *Proc. Phys. Soc.* **86**, 491-500 (1965).

[15] Kelly, H. P., The photoionization cross section for $H_2$from threshold to 30 eV. *Chem. Phys. Lett.* **20**, 547-550 (1973).

[16] Serov V. V., Derbov V. L., Sergeeva T. A. Interpretation of time delay in the ionization of two-center systems. *Phys. Rev. A* **87**, 063414 (2013).

[17] H. Park, & R. N. Zare, Molecular-orbital decomposition of the ionization continuum for a diatomic molecule by angle-and energy-resolved photoelectron spectroscopy. I. Formalism. *J. Chem. Phys.* **104**, 4554-4567 (1996).

[18] G. Breit, & H. A. Bethe, Ingoing Waves in Final State of Scattering Problems. *Phys. Rev.* **93**, 888-890 (1954).

[19] O'Neil, S. V. , & Reinhardt, W. P., Photoionization of molecular hydrogen. *J. Chem. Phys.* **69**, 2126-2142 (1978).

[20] Chung, Y. M., Lee, E., Masuoka, T., & Samson, J. A. R., Dissociative photoionization of H_2 from 18 to 124 eV. *J. Chem. Phys.* **99**, 885-889 (1993).

[21] Nabekawa, Y., Shimizu, T., Okino, T., Furusawa, K., Hasegawa, H., Yamanouchi, K., & Midorikawa, K., Conclusive Evidence of an Attosecond Pulse Train Observed with the Mode-Resolved Autocorrelation Technique. *Phys. Rev. Lett.* **96**, 083901 (2006).
